# Supplementary material for: A pilot study using hospital surveillance and a birth cohort to investigate enteric pathogens and malnutrition in children, Dili, Timor-Leste
Source: PLoS One. 2024 Feb 1;19(2):e0296774. doi: 10.1371/journal.pone.0296774 (PMC10833528; doi:10.1371/journal.pone.0296774)
Supplement: S1 Table — (PDF) [file pone.0296774.s002.pdf]

S1 Table. Weight-for-height, height-for-age, and weight-for-age z-score for birth cohort infants at each home visit in Dili, Timor-Leste, 2019-2020.

	**Weight-for-height z-score (median, IQR)**	**Wasted *n* (%, 95%CI)[§]**	**Height-for-age z-score (median, IQR)**	**Stunted *n* (%, 95%CI)[§]**	**Weight-for-age z-score (median, IQR)**	**Underweight *n* (%, 95%CI)[§]**	**Number of infants (% female)**	**Age in days (mean, ±*SD*)**
**Birth**	0.9 (-0.5 to 1.6)*	2/56* (3.6, 0.6 to 13.4)	-1.2 (-1.7 to -0.5)	14/60 (23.3, 13.8 to 36.3)	-0.5 (-1.3 to 0.3)	2/60 (3.3, 0.6 to 12.5)	56*/60 (47.0)	0.0 (0.0)
**Visit 1**	-1.2 (-1.8 to -0.7)	9/49 (18.4, 9.2 to 32.5)	0.3 (-0.3 to 0.7)	1/49 (2.0, 0.1 to 12.2)	-0.6 (-1.1 to 0.1)	5/49 (10.2, 3.8 to 23.0)	49 (45.0)	46.2 (10.3)
**Visit 2**	-1.3 (-2.0 to -0.1)	9/41 (22.0, 11.1 to 38.0)	0.2 (-0.5 – 0.6)	0/41 (0.0, 0.0 to 10.7)	-0.7 (-1.3 to -0.2)	1/41 (2.4, 0.1 to 14.4)	41 (44.0)	124.2 (12.9)
**Visit 3**	-0.9 (-2.3 to -0.1)	12/39 (30.8, 17.5 to 47.7)	-0.1 (-0.9 – 0.8)	1/39 (2.6, 1.3 to 15.1)	-1.2 (-1.7 to -0.2)	5/39 (12.8, 4.8 to 28.2)	39 (46.0)	216.2 (22.7)
**Visit 4**	-1.0 (-1.9 to -0.1)	8/32 (25.0, 12.1 to 43.8)	-0.3 (-1.3 – 0.4)	3/32 (9.4, 2.5 to 26.2)	-1.2 (-1.6 to -0.2)	2/32 (6.3, 1.1 to 22.2)	32 (47.0)	380.5 (19.2)

* WHZ not calculated for four infants at birth as their length was below lower limit of 45cm for calculation. IQR = interquartile range. *n* = number of cases. 95% CI = 95% confidence interval. *SD* = standard deviation. § wasted, stunted, and underweight includes moderate (≤-2) and severe (≤-3) z-scores.

S2 Table. Adjusted univariate odds ratios using a generalised estimating equations model for differences in pathogens detected between diarrhoeal and non-diarrhoea stool samples for infants from a birth cohort in Dili, Timor-Leste, 2019-2020.

	Solid or semi-solid stool (N=114)	Watery stool with or without blood (N=27)	GEE aOR (95% CI)
Age (adjusted for individual study participant, sex and season)	114	27	
3 months or less	35 (30.7%)	10 (37.0%)	ref
3 to 6 months	26 (22.8%)	11 (40.7%)	2.9 (0.9-9.5)
6 to 9 months	26 (22.8%)	6 (22.2%)	3.3 (0.5-23.4)
9 to 12 months	5 (4.4%)	0 (0.0%)	NA
12 months or more	22 (19.3%)	0 (0.0%)	NA
Sex (adjusted for individual study participant, age and season)	114	27	
Female	50 (43.9%)	15 (55.6%)	ref
Male	64 (56.1%)	12 (44.4%)	0.5 (0.2-1.3)
Season (adjusted for individual study participant, age and sex)	114	27	
Dry (May to November)	71 (62.3%)	18 (66.7%)	ref
Wet (December to April)	43 (37.7%)	9 (33.3%)	0.2 (0.0-1.2)
**All variables adjusted for age, sex, season and individual study participant in GEE model**			
Campylobacter spp.	114	27	
No	102 (89.5%)	24 (88.9%)	ref
Yes	12 (10.5%)	3 (11.1%)	2.6 (0.5-12.4)
C. difficile	114	27	
No	102 (89.5%)	26 (96.3%)	ref
Yes	12 (10.5%)	1 (3.7%)	0.3 (0.0-2.2)
Plesiomonas	114	27	
No	113 (99.1%)	27 (100.0%)	
Yes	1 (0.9%)	0 (0.0%)	NA
Salmonella spp.	114	27	
No	110 (96.5%)	27 (100.0%)	
Yes	4 (3.5%)	0 (0.0%)	NA
Vibrio spp.	114	27	
No	111 (97.4%)	27 (100.0%)	
Yes	3 (2.6%)	0 (0.0%)	NA
Vibrio cholerae	114	27	
No	112 (98.2%)	27 (100.0%)	
Yes	2 (1.8%)	0 (0.0%)	NA
EAEC	114	27	
No	52 (45.6%)	12 (44.4%)	ref
Yes	62 (54.4%)	15 (55.6%)	1.6 (0.6-4.2)
EPEC	114	27	
No	73 (64.0%)	19 (70.4%)	ref
Yes	41 (36.0%)	8 (29.6%)	1.7 (0.6-5.3)
ETEC	114	27	
No	97 (85.1%)	22 (81.5%)	ref
Yes	17 (14.9%)	5 (18.5%)	2.0 (0.5-7.3)
STEC	114	27	
No	112 (98.2%)	27 (100.0%)	ref
Yes	2 (1.8%)	0 (0.0%)	1.5 (0.3-7.1)

E. coli O 157		114	27
No	114 (100.0%)	27 (100.0%)	NA
Shigella /EIEC		114	27
No	113 (99.1%)	26 (96.3%)	ref
Yes	1 (0.9%)	1 (3.7%)	3.0 (0.2-46.0)
Cryptosporidium		114	27
No	111 (97.4%)	27 (100.0%)	ref
Yes	3 (2.6%)	0 (0.0%)	1.2 (0.3-4.0)
Cyclospora		114	27
No	114 (100.0%)	27 (100.0%)	NA
Entamoeba		114	27
No	114 (100.0%)	27 (100.0%)	NA
Giardia		114	27
No	114 (100.0%)	26 (96.3%)	
Yes	0 (0.0%)	1 (3.7%)	NA
Adenovirus		114	27
No	108 (94.7%)	27 (100.0%)	
Yes	6 (5.3%)	0 (0.0%)	NA
Astrovirus		114	27
No	114 (100.0%)	27 (100.0%)	NA
Norovirus		114	27
No	104 (91.2%)	25 (92.6%)	ref
Yes	10 (8.8%)	2 (7.4%)	1.5 (0.2-9.2)
Rotavirus		114	27
No	108 (94.7%)	24 (88.9%)	ref
Yes	6 (5.3%)	3 (11.1%)	3.8 (0.4-35.5)
Sapovirus		114	27
No	111 (97.4%)	26 (96.3%)	ref
Yes	3 (2.6%)	1 (3.7%)	1.5 (0.1-15.8)

S3 Table. Adjusted univariate odds ratios using a generalised estimating equations model for risk factors associated with pathogen detection for infants in a birth cohort, Dili, Timor-Leste, 2019-2020.

	Pathogen detected (N=97)	No pathogen detected (N=44)	GEE aOR (95% CI)
Age (adjusted for individual study participant and season)	97	44	
Less than 3 months	16 (16.5%)	29 (65.9%)	ref
3 to 6 months	27 (27.8%)	9 (20.5%)	2.8 (0.9-8.8)
6 to 9 months	30 (30.9%)	3 (6.8%)	3.6 (0.4-32.1)
9 to 12 months	4 (4.1%)	0 (0.0%)	NA
Over 12 months	20 (20.6%)	3 (6.8%)	12.1 (3.1-47.0) *
Season (adjusted for individual study participant and age)	97	44	
Dry (May to November)	50 (51.5%)	39 (88.6%)	ref
Wet (December to April)	47 (48.5%)	5 (11.4%)	5.1 (0.9-29.3)
**All variables adjusted for age, season and individual study participant in GEE model**			
Sex of child	97	44	
Female	43 (44.3%)	22 (50.0%)	ref
Male	54 (55.7%)	22 (50.0%)	1.5 (0.7-3.5)
Household size	97	44	
5 or less	14 (14.4%)	9 (20.5%)	0.6 (0.2-2.1)
6 to 10	61 (62.9%)	24 (54.5%)	ref
11 to 15	20 (20.6%)	10 (22.7%)	1.0 (0.4-2.8)
16 or more	2 (2.1%)	1 (2.3%)	0.4 (0.0-11.9)
Highest level of education by primary carer	96	44	
Finished year 12 and/or further study	60 (62.5%)	30 (68.2%)	ref
Did not complete schooling or finished before year 12	36 (37.5%)	14 (31.8%)	1.0 (0.4-2.5)

Primary feeding type of child	96	44		
Breastfed	31 (32.3%)	26 (59.1%)	ref	
Bottlefed	16 (16.7%)	1 (2.3%)	8.3 (1.1-62.7)	*
Combined breast and bottle	22 (22.9%)	14 (31.8%)	1.6 (0.6-4.4)	
Combined plus solid	27 (28.1%)	3 (6.8%)	0.9 (0.2-3.8)	
How is the bottle cleaned?	54	16		
Cleaned	41 (75.9%)	15 (93.8%)	ref	
Rinse only	13 (24.1%)	1 (6.2%)	0.9 (0.1-6.9)	
Where is food prepared?	96	44		
Separate area (building or outdoors)	47 (48.9%)	19 (43.2%)	ref	
House	49 (51.0%)	25 (56.8%)	0.7 (0.3-1.7)	
Is there a separate room in the house for kitchen?	49	25		
Yes	41 (83.7%)	21 (84.0%)	ref	
No	8 (16.3%)	4 (16.0%)	1.0 (0.2-4.6)	
How is garbage disposed?	97	44		
Garbage bin or dumpster	78 (80.4%)	31 (70.5%)	ref	
Buried	2 (2.1%)	0 (0.0%)	NA	
Burned	7 (7.2%)	9 (20.5%)	1.0 (0.3-3.3)	
Thrown away	10 (10.3%)	4 (9.1%)	0.5 (0.1-3.4)	
What toilet facilities are available?	97	44		
Latrine (pit or slab)	94 (96.9%)	43 (97.7%)	ref	
Flush toilet	3 (3.1%)	1 (2.3%)	0.4 (0.0-5.2)	
What is the main water source for drinking water?	97	44		
Municipal supply	62 (63.9%)	35 (79.5%)	ref	
Bore or ground	16 (16.5%)	6 (13.6%)	2.4 (0.7-7.8)	
Bottled	19 (19.6%)	3 (6.8%)	0.7 (0.1-3.1)	

What is the main source for washing water?		95	43
Municipal	74 (77.9%)	35 (81.4%)	ref
Bore or ground	20 (21.1%)	7 (16.3%)	2.1 (0.7-6.1)
Bottled	1 (1.1%)	1 (2.3%)	0.1 (0.0-1.7)
Do you store water?		97	44
Yes	90 (92.8%)	43 (97.7%)	ref
No	7 (7.2%)	1 (2.3%)	0.7 (0.1-7.2)
Is the stored water covered?		67	41
Always covered	12 (17.9%)	5 (12.2%)	ref
Mostly or sometimes covered	55 (82.1%)	36 (87.8%)	0.3 (0.1-1.0)
Do you treat water before use?		97	44
Yes	70 (72.2%)	40 (90.9%)	ref
No	27 (27.8%)	4 (9.1%)	0.4 (0.1-3.0)
How many animals do you have?		72	34
None	1 (1.4%)	1 (2.9%)	0.5 (0.0-4.7)
Less than 5	45 (62.5%)	17 (50.0%)	1.5 (0.5-4.4)
5 to 10	19 (26.4%)	11 (32.4%)	ref
More than 10	7 (9.7%)	5 (14.7%)	0.6 (0.1-3.6)
How many farm animals (e.g., goats, chickens, cows) do you have?		72	34
None	15 (20.8%)	6 (17.6%)	ref
Less than 5	34 (47.2%)	16 (47.1%)	1.0 (0.3-3.4)
5 to 10	17 (23.6%)	10 (29.4%)	0.6 (0.2-2.3)
More than 10	6 (8.3%)	2 (5.9%)	0.6 (0.0-10.5)
How many pet animals (e.g., dogs, cats) do you have?		72	34
None	33 (45.8%)	14 (41.2%)	ref
Less than 5	37 (51.4%)	17 (50.0%)	0.8 (0.3-2.1)
5 to 10	2 (2.8%)	3 (8.8%)	0.5 (0.0-12.5)

Where are these animals located?	68	33	
Free outside	17 (25.0%)	11 (33.3%)	ref
Cage or pen	11 (16.2%)	0 (0.0%)	NA
Free inside	29 (42.6%)	15 (45.5%)	1.5 (0.4-5.0)
Tied up	11 (16.2%)	7 (21.2%)	1.7 (0.4-8.1)

S4 Table. Adjusted univariate odds ratios using a generalised estimating equations model for risk factors associated with moderate wasting for infants in a birth cohort, Dili, Timor-Leste, 2019-2020.

	Under -2 WHZ score (N=38)	Over -2 WHZ score (N=123)	GEE aOR (95% CI)	
Age (adjusted for individual study participant and season)	38	123		
Less than 3 months	9 (23.7%)	40 (32.5%)	ref	
3 to 6 months	10 (26.3%)	33 (26.8%)	2.8 (0.9-8.6)	
6 to 9 months	11 (28.9%)	25 (20.3%)	12.3 (1.7-87.6)	*
9 to 12 months	1 (2.6%)	6 (4.9%)	0.9 (0.1-8.1)	
Over 12 months	7 (18.4%)	19 (15.4%)	1.6 (0.5-5.1)	
Season (adjusted for individual study participant and age)	38	123		
Dry (May to November)	25 (65.8%)	77 (62.6%)	ref	
Wet (December to April)	13 (34.2%)	46 (37.4%)	0.2 (0.0-0.9)	*
**All variables adjusted for age, season and individual study participant in GEE model**				
Sex of child	38	123		
Female	19 (50.0%)	54 (43.9%)	ref	
Male	19 (50.0%)	69 (56.1%)	0.8 (0.4-1.7)	
Household size	38	123		
5 or less	2 (5.3%)	25 (20.3%)	0.3 (0.1-1.1)	
6 to 10	25 (65.8%)	71 (57.7%)	ref	
11 to 15	10 (26.3%)	25 (20.3%)	1.2 (0.5-2.9)	
16 or more	1 (2.6%)	2 (1.6%)	1.0 (0.1-9.7)	
Highest level of education by primary carer	38	121		
Finished year 12 and/or further study	28 (73.7%)	79 (65.3%)	ref	
Did not complete schooling or finished before year 12	10 (26.3%)	42 (34.7%)	0.7 (0.3-1.5)	

Primary feeding type of child	38	122	
Breastfed	12 (31.6%)	55 (45.1%)	ref
Bottlefed	4 (10.5%)	15 (12.3%)	1.2 (0.3-4.8)
Combined breast and bottle	11 (28.9%)	31 (25.4%)	1.6 (0.6-4.0)
Combined plus solid	11 (28.9%)	21 (17.2%)	5.9 (0.4-83.7)
How is the bottle cleaned?	20	59	
Cleaned	15 (75.0%)	44 (74.6%)	ref
Rinse only	5 (25.0%)	15 (25.4%)	2.9 (0.4-20.1)
Where is food prepared?	122	38	
Separate area (building or outdoors)	58 (47.5%)	20 (52.6%)	ref
House	64 (52.5%)	18 (47.4%)	0.7 (0.3-1.6)
Is there a separate room in the house for kitchen?	18	64	
Yes	14 (77.8%)	55 (85.9%)	ref
No	4 (22.2%)	9 (14.1%)	1.6 (0.4-5.9)
How is garbage disposed?	38	123	
Garbage bin or dumpster	29 (76.3%)	96 (78.0%)	ref
Buried	1 (2.6%)	2 (1.6%)	2.8 (0.3-31.1)
Burned	2 (5.3%)	15 (12.2%)	0.5 (0.1-3.0)
Thrown away	6 (15.8%)	10 (8.1%)	2.6 (0.7-10.4)
What toilet facilities are available?	38	123	
Latrine	38 (100.0%)	119 (96.7%)	
Flush	0 (0.0%)	4 (3.3%)	NA
What is the main water source for drinking water?	38	123	
Municipal supply	22 (57.9%)	84 (68.3%)	ref
Bore or ground	6 (15.8%)	20 (16.3%)	1.7 (0.5-5.1)
Bottled	10 (26.3%)	19 (15.4%)	2.5 (0.9-7.1)

What is the main source for washing water?	35	122	
Municipal	29 (82.9%)	95 (77.9%)	ref
Bore or ground	5 (14.3%)	26 (21.3%)	0.8 (0.3-2.3)
Bottled	1 (2.9%)	1 (0.8%)	2.6 (0.1-46.7)
Do you store water?	38	123	
Yes	35 (92.1%)	118 (95.9%)	ref
No	3 (7.9%)	5 (4.1%)	1.5 (0.3-7.4)
Is the stored water covered?	27	94	
Always covered	4 (14.8%)	14 (14.9%)	ref
Mostly or sometimes covered	23 (85.2%)	80 (85.1%)	0.5 (0.1-1.8)
Do you treat water before use?	38	123	
Yes	28 (73.7%)	96 (78.0%)	ref
No	10 (26.3%)	27 (22.0%)	1.2 (0.3-4.6)
How many animals do you have?	29	93	
None	0 (0.0%)	2 (2.2%)	NA
Less than 5	20 (69.0%)	53 (57.0%)	0.8 (0.3-2.2)
5 to 10	8 (27.6%)	27 (29.0%)	ref
More than 10	1 (3.4%)	11 (11.8%)	0.3 (0.0-2.5)
How many farm animals (i.e., goats, chickens, cows) do you have?	29	93	
None	6 (20.7%)	19 (20.4%)	ref
Less than 5	14 (48.3%)	44 (47.3%)	1.1 (0.4-3.2)
5 to 10	9 (31.0%)	22 (23.7%)	2.0 (0.6-6.9)
More than 10	0 (0.0%)	8 (8.6%)	NA
How many pet animals (i.e., dogs, cats) do you have?	29	93	
None	11 (37.9%)	41 (44.1%)	ref
Less than 5	16 (55.2%)	48 (51.6%)	1.3 (0.5-3.4)
5 to 10	2 (6.9%)	4 (4.3%)	3.8 (0.6-23.5)

Where are these animals located?	28	89	
Free outside	11 (39.3%)	21 (23.6%)	ref
Cage/Pen	4 (14.3%)	10 (11.2%)	0.6 (0.1-2.5)
Free inside	10 (35.7%)	40 (44.9%)	0.6 (0.2-1.9)
Tied up	3 (10.7%)	18 (20.2%)	0.4 (0.1-1.9)

S5 Table. Weight-for-height, height-for-age, and weight-for-age, and MUAC-for-age for hospital cohort children at admission, Dili, Timor-Leste, 2019-2020.

	**Weight-for-height z-score (median, IQR)**	**Wasted** ***n*** **(%, 95%CI)§**	**Height-for-age z-score (median, IQR)**	**Stunted** ***n*** **(%, 95%CI)§**	**Weight-for-age z-score (median, IQR)**	**Underweight** ***n*** **(%, 95%CI)§**	**MUAC-for-age z-score (median, IQR)**	**Acutely malnourished** ***n*** **(%, 95%CI)§**	**Number of children (% female)**	**Age in months (mean, ±*SD*)**
**All**	-3.7 (-4.7 to -2.9)	135/153* (88.2, 81.8 to 92.7)	-2.5 (-3.5 to -1.1)	97/156* (62.2, 54.0 to 69.7)	-3.8 (-4.6 to -3.1)	141/157* (89.8, 83.7 to 93.9)	-3.5 (-4.3 to -2.9)	130/144* (90.3, 83.9 to 94.4)	157/159* (52.9)	17.7 (11.4)
**SAM**	-3.9 (-4.7 to -3.0)	124/134* (92.5, 86.4 to 96.2)	-2.6 (-3.6 to -1.4)	88/137* (64.2, 55.5 to 72.1)	-3.9 (-4.8 to -3.2)	128/138* (92.8, 86.7 to 96.3)	-3.7 (-4.3 to -3.2)	120/128* (93.8, 87.7 to 97.1)	138/140* (54.3)	17.5 (10.8)
**Severe diarrhoea**	-2.6 (-4.2 to -1.1)	11/19 (57.9, 34.0 to 78.9)	-1.4 (-2.8 to -0.5)	9/19 (47.4, 25.2 to 70.5)	-3.0 (-3.7 to -1.4)	13/19 (68.4, 43.5 to 86.4)	-2.3 (-2.9 to -1.0)	10/16* (62.5, 35.9 to 83.7)	19 (42.1)	18.9 (15.6)

* calculations do not include all 159 children due to missing data or having measurements below the lower calculation thresholds. SAM = severe acute malnutrition. IQR = interquartile range. *n* = number of cases. 95% CI = 95% confidence interval. *SD* = standard deviation. MUAC = middle-upper arm circumference. § wasted, stunted, underweight, and acutely malnourished includes moderate (≤-2) and severe (≤-3) z-scores.

S6 Table. Mean age in months at admission by diagnosis and type of enteric pathogen detected for hospital-based surveillance cases, Dili, Timor-Leste, 2019–2020.

Enteric pathogen type	All hospitalised cases (mean age in months, ±*SD*)	SAM cases (mean age in months, ±*SD*)	Severe diarrhoea (mean age in months, ±*SD*)
**Bacteria***	17.8 (11.2)	17.5 (10.6)	19.8 (14.8)
**Parasites†^**	19.3 (11.7)	18.8 (11.4)	25.0 (14.9)
**Viruses‡**	13.8 (8.1)	14.0 (8.5)	12.5 (5.1)

SD = standard deviation. SAM = severe acute malnutrition. *Bacteria include *Campylobacter* spp., *Clostridioides difficile, Plesiomonas shigelloides, Salmonella* spp., *Vibrio* spp., diarrhoeagenic *Escherichia coli,* and *Shigella.* †Parasites include *Cryptosporidium* spp., *Giardia lamblia*, *Cyclospora cayetanensis*, and *Entamoeba histolytica*. ‡Viruses include adenovirus, astrovirus, norovirus, rotavirus, and sapovirus. ^ indicates p value < 0.05 between SAM and severe diarrhoea diagnosis groups.

S7 Table. Adjusted univariate odds ratios using a generalised linear model for differences in pathogens detected between diarrhoeal and non-diarrhoea stool samples for children from a hospital-based surveillance cohort in Dili, Timor-Leste, 2019-2020.

	Solid or semi-solid stool (N=136)	Watery stool with or without blood (N=11)	aOR (95% CI)	
Age (adjusted for sex and season)	136	11		
0 to 12 months	52 (38.2%)	3 (27.3%)	ref	
1 to 2 years	56 (41.2%)	5 (45.5%)	1.6 (0.4-8.5)	
2 to 3 years	14 (10.3%)	2 (18.2%)	2.6 (0.3-18.0)	
3 to 4 years	11 (8.1%)	1 (9.1%)	1.8 (0.1-16.0)	
4 to 5 years	3 (2.2%)	0 (0.0%)	NA	
Sex (adjusted for age and season)	136	11		
Female	71 (52.2%)	7 (63.6%)	ref	
Male	65 (47.8%)	4 (36.4%)	0.6 (0.2-2.2)	
Season (adjusted for age and sex)	136	11		
Dry (May to November)	40 (29.4%)	3 (27.3%)	ref	
Wet (December to April)	96 (70.6%)	8 (72.7%)	0.9 (0.2-4.5)	
**All variables adjusted for age, sex, season in generalised linear model**				
Campylobacter spp.	136	11		
No	104 (76.5%)	7 (63.6%)	ref	
Yes	32 (23.5%)	4 (36.4%)	1.8 (0.4-6.8)	
C. difficile	136	11		
No	131 (96.3%)	11 (100.0%)		
Yes	5 (3.7%)	0 (0.0%)	NA	
Plesiomonas	136	11		
No	135 (99.3%)	11 (100.0%)		
Yes	1 (0.7%)	0 (0.0%)	NA	
Salmonella spp.	136	11		
No	133 (97.8%)	9 (81.8%)	ref	
Yes	3 (2.2%)	2 (18.2%)	17.2 (1.6-201.3)	*
Vibrio spp.	136	11		
No	131 (96.3%)	11 (100.0%)		
Yes	5 (3.7%)	0 (0.0%)	NA	
Vibrio cholerae	136	11		
No	133 (97.8%)	11 (100.0%)		
Yes	3 (2.2%)	0 (0.0%)	NA	
EAEC	136	11		
No	49 (36.0%)	5 (45.5%)	ref	
Yes	87 (64.0%)	6 (54.5%)	0.6 (0.2-2.3)	
EPEC	136	11		
No	63 (46.3%)	4 (36.4%)	ref	
Yes	73 (53.7%)	7 (63.6%)	1.5 (0.4-6.8)	
ETEC	136	11		
No	94 (69.1%)	8 (72.7%)	ref	
Yes	42 (30.9%)	3 (27.3%)	0.7 (0.1-2.9)	
STEC	136	11		
No	132 (97.1%)	11 (100.0%)		
Yes	4 (2.9%)	0 (0.0%)	NA	
E. coli O157	136	11		
No	134 (98.5%)	11 (100.0%)		
Yes	2 (1.5%)	0 (0.0%)	NA	

Shigella /EIEC		136		11		
No	111 (81.6%)		6 (54.5%)		ref	
Yes	25 (18.4%)		5 (45.5%)		3.8 (1.0-14.4)	*
Cryptosporidium		136		11		
No	121 (89.0%)		8 (72.7%)		ref	
Yes	15 (11.0%)		3 (27.3%)		3.0 (0.6-12.3)	
Cyclospora		136		11		
No	125 (91.9%)		10 (90.9%)		ref	
Yes	11 (8.1%)		1 (9.1%)		0.9 (0.0-6.0)	
Entamoeba		136		11		
No	135 (99.3%)		11 (100.0%)			
Yes	1 (0.7%)		0 (0.0%)		NA	
Giardia		136		11		
No	107 (78.7%)		7 (63.6%)		ref	
Yes	29 (21.3%)		4 (36.4%)		2.2 (0.5-8.7)	
Adenovirus		136		11		
No	133 (97.8%)		10 (90.9%)		ref	
Yes	3 (2.2%)		1 (9.1%)		5.5 (0.2-58.7)	
Astrovirus		136		11		
No	129 (94.9%)		11 (100.0%)			
Yes	7 (5.1%)		0 (0.0%)		NA	
Norovirus		136		11		
No	124 (91.2%)		10 (90.9%)		ref	
Yes	12 (8.8%)		1 (9.1%)		1.0 (0.1-6.0)	
Rotavirus		136		11		
No	130 (95.6%)		10 (90.9%)		ref	
Yes	6 (4.4%)		1 (9.1%)		2.7 (0.1-24.7)	
Sapovirus		136		11		
No	123 (90.4%)		10 (90.9%)		ref	
Yes	13 (9.6%)		1 (9.1%)		0.9 (0.0-6.0)	

S1 Fig. Total pathogen detections for a birth cohort and hospital surveillance cohort, stratified by age group, in infants and children in Dili, Timor-Leste, 2019–2020.

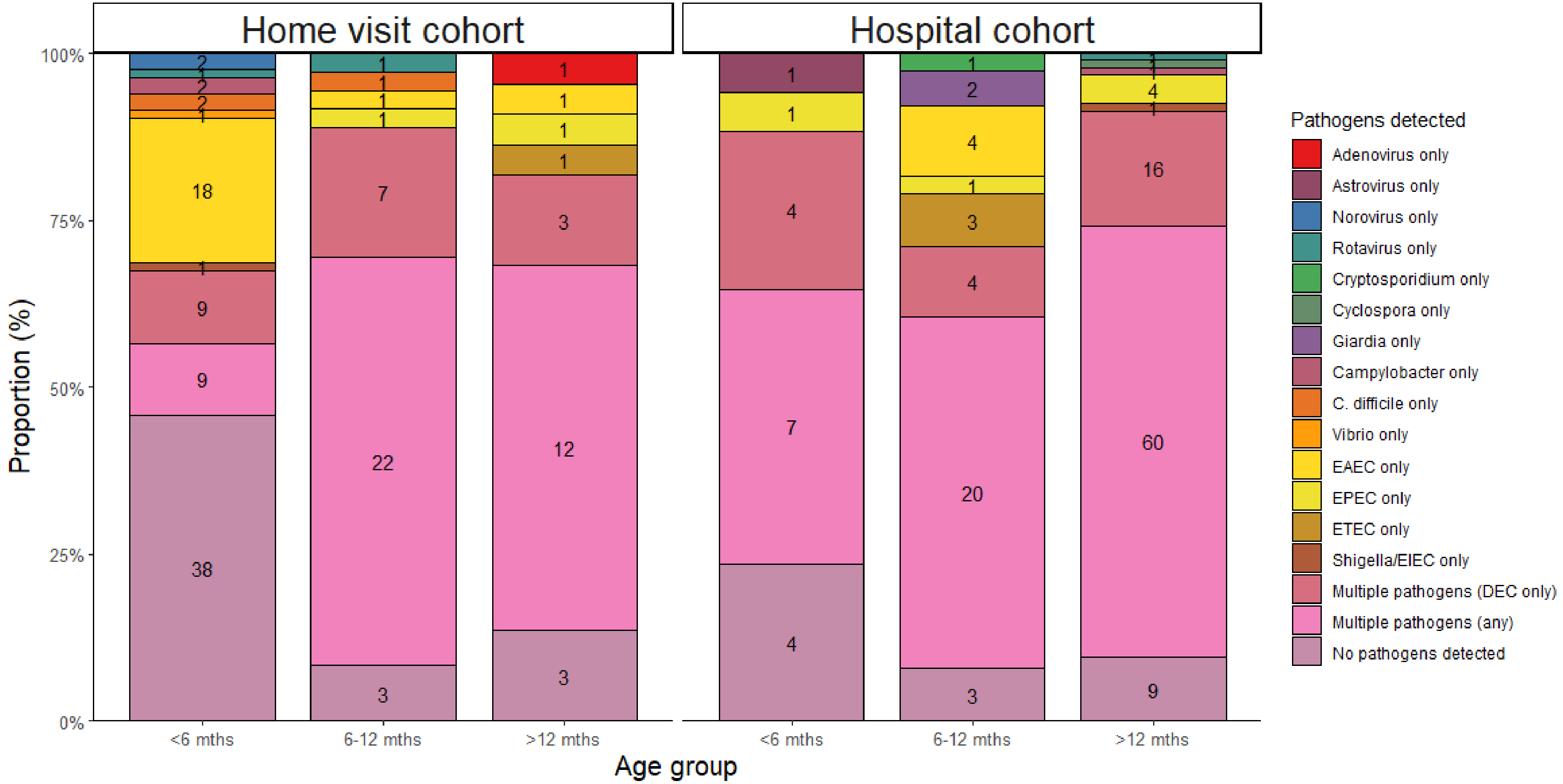

S1 File. Questionnaire administered to families during birth cohort home visits in Dili, Timor-Leste, 2019–2020.

FAMILY QUESTIONNAIRE (PARENT/CAREGIVER)

Stunting and gastrointestinal infections in infants in Timor-Leste: A pilot study

Attached is a paper version of the electronic questionnaire that will be administered by researchers to the parent or guardian of the participating infant at each of the four home visits.

The questionnaire will be created using the secure Research Electronic Data Capture (REDCap) software https://projectredcap.org/software/.

The researcher will ask all the questions and enter the data directly into the questionnaire on a laptop or tablet, on return to the office they will upload it to the database and the data will be erased from the laptop/tablet.

Most of the questions are copied verbatim from the Timor-Leste Demographic and Health Survey 2016. These questions were tested and validated prior to the survey. Other questions are adapted from a previously approved study: *Should integrated deworming and WASH programs for STH control be delivered in schools or the community? – a pilot study in preparation of a cluster randomized trial* GECQ Reference Number: GECQ/EP/2015/025

FAMILY QUESTIONNAIRE (PARENT/CAREGIVER)

Stunting and gastrointestinal infections in infants in Timor-Leste: A pilot study

Address:

Date : ☐☐ / ☐☐ / 2 0 ☐☐ Interviewer initials: ☐☐

Consent checklist

Has written consent been obtained? Y ☐ N ☐ *Only proceed if yes*

A. Baby details

1. Participant ID: ☐☐☐☐☐☐☐☐

2. Name: ☐

3. Gender: M ☐ F ☐

4. Date of Birth (DD/MM/YYYY): ______________________

5. Name of caregiver: ☐

6. Relationship with participant ☐

7. How many people of the following age groups live in your household?

<5 years	
5-17 years	
18-65 years	
>65 years	
Total	

B. Family Health

9. To your knowledge, has your baby taken antibiotic medicine since birth/our last visit? Y ☐ N ☐ Refused ☐

10. In the last 24 hours, how many bowel motions has your baby passed? ☐

11. In the past 24 hours, describe your baby's bowel motions:
(Tick ONLY ONE correct response)
- Normal ☐
- Loose and/or watery ☐
- Loose and/or watery with blood ☐

12. In the last two weeks has your baby had loose stools 3 or more times in one day? Y ☐ N ☐

If yes, was there any visible blood in his/her stools? Y ☐ N ☐

13. In the last 24 hours have you, or anyone else living with you (other than the baby), had loose stools 3 or more times? Y ☐ N ☐

If yes, was there any visible blood in his/her stools? Y ☐ N ☐

C. Food preparation

14. How is your baby fed?

Tick (√) all items mentioned WITHOUT reading out options:

Breast-fed			
Bottle-fed		Specify:	
Solid foods		Specify:	
Other		Specify:	
Don't know			
Refused			

15. If bottle-fed, how do you clean the bottle between feeds?

Tick (√) all items mentioned WITHOUT reading out options:

Rinse with water			
Wash with soap			
Boil			
Other		Specify:	
Don't know			
Refused			

16. Is the cooking for the family usually done in the house, in a separate building, or outdoors?

Tick (√) one item only:

In the house		Go to 17	
In a separate building			
Outdoors under cover			
Outdoors			
Other		Specify:	
Don't know			
Refused			

17. Do you have a separate room which is used as a kitchen? Y ☐ N ☐ Refused ☐

D. Sanitation data

18. How do you usually dispose of your household garbage?

Tick (√) one item only:

Burned			
Buried			
Thrown away			
Garbage bin/dumpster and picked up			
Recycled			
Other		Specify:	
Don't know			
Refused			

19. What kind of toilet facility do members of your household usually use?

Tick off (√) toilet type, using picture sheet of different toilet types as a guide:

Flush to septic tank			
Flush to pit latrine			
Flush to somewhere else		Specify:	
Flush don't know where			
Ventilated improved pit latrine			

Pit latrine with slab			
Pit latrine without slab/open pit			
Composting toilet			
Bucket toilet			
Hanging toilet or latrine			
No facility/bush/field			
Other		Specify:	
Don’t know			
Refused			

20. What are the sources of drinking water for your household?

Read all options and tick (√) all items mentioned. Show the picture sheet of different water sources. Observe sources if possible:

Piped water into dwelling (inside house)			
Piped water to yard/plot - this house only			
Piped water shared with other houses (to yard/plot or to communal place)			
Public tap/standpipe			
Tubewell or borehole or protected dug well			
Unprotected dug well			
Protected spring			
Unprotected spring			
Rainwater			
Tanker truck			
Surface water			
Bottled water			
Other		Specify:	
Don’t know			
Refused			

21. What are the sources of water used by your household for other purposes such as cooking and handwashing?

Read all options and tick (√) all items mentioned. Show the picture sheet of different water sources. Observe source if possible:

Piped water into dwelling (inside house)			
Piped water to yard/plot - this house only			
Piped water shared with other houses (to yard/plot or to communal place)			
Public tap/standpipe			
Tubewell or borehole or protected dug well			
Unprotected dug well			
Protected spring			
Unprotected spring			
Rainwater			
Tanker truck			
Surface water			
Bottled water			
Other		Specify:	
Don’t know			
Refused			

22. Where is the main water source located?

CHOOSE ONLY ONE OPTION with a tick (√):

In your house	
In your yard or plot	

Other		Specify:	
Don’t know			
Refused			

23. Do you store water from this main source in the household? Y ☐ N ☐ *If no, skip to question 25.*

24. If yes, what type of container(s) do you use to store water?

Tick all items mentioned or demonstrated:

Jerry-can		Covered?	All		Some		None			
Balde/basin		Covered?	All		Some		None			
Ceramic pot		Covered?	All		Some		None			
Other		Covered?	All		Some		None		Specify:	
Don’t know										
Refused										

25. Do you do anything to your water to make it safer? Y ☐ N ☐ *If no, skip to question 28.*

26. If yes, which purposes would you treat the water for?

Read all options and tick (√) all items mentioned:

Drinking			
Cooking			
Dishwashing			
Hand/body washing			
Other		Specify:	
Don’t know			
Refused			

27. What do you treat it with? *Read all options and tick (√) all items mentioned:*

Household bleach			
Boil			
Strain through cloth			
Filter			
Other		Specify:	
Don’t know			
Refused			

28. What is your highest level of education? *Tick only one option.*

Never went to school	
Not finished primary school (6 years)	
Completed primary school	
Not finished pre-secundario (9 years)	
Completed pre-secundario (9 years)	
Not finished secundario (12 years)	
Completed secundario (12 years)	
Completed professional training	
Not finished university	
Completed university	
Don’t know	
Refused	

29. What is your current employment status?

Employed/has job		→ Specify MAIN job:		
Doing housework				
Student				

Retired	
Long-term disabled	
Unemployed	
Don’t know	
Refused	

30. How much income did your household receive over the last year?

Less than USD $365	
USD $365-730	
USD $730-1460	
More than USD $1460	
Don’t know	
Refused	

31. Does your household keep any of the following animals?

Dogs?	Y		N		If yes, how many?		Where are they kept?	
Pigs?	Y		N		If yes, how many?		Where are they kept?	
Chickens?	Y		N		If yes, how many?		Where are they kept?	
Cows/buffalo?	Y		N		If yes, how many?		Where are they kept?	
Other animals?	Y		N		If yes, which and how many?		Where are they kept?	
Don’t know								
Refused								

32. Observe presence of animals moving freely inside or around the house. Select all that apply.

Animals in pens/cages	
Animals moving freely outside	
Animals moving freely inside	

This completes the questionnaire. We are grateful for your participation - thank you.

Inclusivity in global research

PLOS' policy on inclusivity in global research aims to improve transparency in the reporting of research performed outside of researchers' own country or community and ensures that PLOS publications reporting global research adhere to high standards for research ethics and authorship. Authors of relevant research articles may be asked to complete the questionnaire below, which outlines ethical, cultural, and scientific considerations specific to inclusivity in global research. This questionnaire may be requested when researchers have travelled to a different country to conduct research, if research uses samples collected in another country, research with Indigenous populations or their lands, or if research is on cultural artefacts. Researchers travelling to another country solely to use laboratory equipment will not normally be required to complete the questionnaire. However, the questionnaire can be requested at the journal's discretion for any submission – if you have been requested to complete this questionnaire by the PLOS journal you submitted to, please do so.

Please complete the questionnaire below and include this as a Supporting Information file with your manuscript. Note that if your paper is accepted for publication, this checklist will be published with your article in the supporting information files. Please ensure that you reference the checklist in the main body of your manuscript. We suggest adding a subsection 'Inclusivity in global research' to your Methods section and adding the following sentence: "Additional information regarding the ethical, cultural, and scientific considerations specific to inclusivity in global research is included in the Supporting Information (SX Checklist)"

The questions have been designed to be applicable to a wide range of study types, and there are subsections for both human subjects research and non-human subjects research. If any of the questions are not relevant to your research please mark them as "N/A" as appropriate.

Ethical considerations, permits and authorship

This section is applicable to all research types.

Provide details as to who granted permissions and/or consent for the study to take place in the Methods section of your manuscript. This should include the names of **all** ethics boards, governmental organizations, community leaders or other bodies that provided approval for the study. If individuals provided approval refer to these people by their role or title but do not list their name(s).

Reported on page number: 10

If there were any deviations from the study protocol after approval was obtained please provide details of these changes in the Methods section of your manuscript.

Reported on page number: 6

Did this study involve local collaborators that are residents of the country where the research was conducted or members of the community studied? If you do not have any authors from said communities, please provide an explanation for this below.

Yes, Timorese residents were included in the study team and are included as both authors of the paper and in the acknowledgements, where appropriate.

Everyone listed as an author should meet PLOS' criteria for authorship and all individuals who meet these criteria should be included in the author byline, rather than the acknowledgements. For further information please see the journal's Authorship Policy.

Human subjects research (e.g. health research, medical research, cross-cultural psychology)

Did you obtain written informed consent from a representative of the local community or region before the research took place? How did you establish who speaks for the community? Details of written informed consent obtained from study participants should be reported separately in the Methods section of your manuscript.

Written consent was sought from individual parents and families, where relevant, for our research. We consulted with senior staff the Ministry of Health in Timor-Leste, Hospital Nacional Guide Valadares, the National Health Laboratory, and the Diagnostic Laboratory for Animal Health throughout the study. Seeking permission from a community leader was not relevant. Details on consent processes are included in our Methods section.

How did members of the local community provide input on the aims of the research investigation, its methodology, and its anticipated outcome(s)?

We consulted and collaborated with local staff from the Ministry of Health, Hospital Nacional Guido Valadares, the National Health Laboratory, and the Diagnostic Laboratory for Animal Health, all in Timor-Leste, to prepare the research aims, methodology, and anticipated outcomes of this study.

When engaging with the local community, how did you ensure that the informed consent documents and other materials could be understood by local stakeholders?

We translated all relevant study documents into the local language, Tetum, for participants to understand. This includes the family questionnaire, written consent form, participant information sheet, faecal sample collection guidelines, and study process overview. Study team members engaging with participants were fluent in Tetum.

Will the findings of the research be made available in an understandable format to stakeholders in the community where the study was conducted (e.g. via a presentation, summary report, copies of publications, etc.)? Please provide details of how this will be achieved.

Yes. Results are delivered to study participants through feedback, workshops, and reports by local team members.

Non-human subjects research using specimens/ animals collected as part of the study, or those housed in archival collections. Examples include archaeology, paleontology, botany and zoology.

Did the permission you obtained from a local authority to perform the study include an agreement on access to outputs and benefit sharing? This may include procedures to enable fair distribution of the benefits and resources arising from the research performed. Please include any details of Prior Informed Consent and Benefit Sharing Agreements obtained. These may be required by field-specific regulations, for example the Convention on Biological Diversity (CBD) and the associated Nagoya Protocol.

N/A

If the material used in your study was imported, please A) provide the year it was imported and B) indicate whether permits were obtained to import/export the materials used, C) provide details of any permits obtained. If this information is not available, please indicate this.

N/A

If you used archival specimens, please state how the material used in your study was acquired by the institute it is held in and provide details of any permits obtained for the original excavations/ sample collection. If this information is not available, please indicate this.

N/A

How was the potential cultural significance of the materials collected in your study to local communities considered in your research design? Were Indigenous peoples and/or local researchers and institutions involved with archaeological excavations / collection of specimens? If so, please provide a description of their involvement.

N/A

If your manuscript includes photographs of human remains please indicate whether authors obtained permission from descendants or affiliated cultural communities to do so.

N/A
