# Supplementary material for: A pilot study using hospital surveillance and a birth cohort to investigate enteric pathogens and malnutrition in children, Dili, Timor-Leste
Source: PLoS One. 2024 Feb 1;19(2):e0296774. doi: 10.1371/journal.pone.0296774 (PMC10833528; doi:10.1371/journal.pone.0296774)
Supplement: S2 Table — * Indicates statistical significance (p value <0.05). 95% CI = 95% confidence interval. ref = reference variable. NA = odds ratio not calculated. (PDF) [file pone.0296774.s003.pdf]

S2 Table. Adjusted univariate odds ratios using a generalised estimating equations model for differences in pathogens detected between diarrhoeal and non-diarrhoea stool samples for infants from a birth cohort in Dili, Timor-Leste, 2019-2020.

	Solid or semi-solid stool (N=114)	Watery stool with or without blood (N=27)	GEE aOR (95% CI)
Age (adjusted for individual study participant, sex and season)	114	27	
3 months or less	35 (30.7%)	10 (37.0%)	ref
3 to 6 months	26 (22.8%)	11 (40.7%)	2.9 (0.9-9.5)
6 to 9 months	26 (22.8%)	6 (22.2%)	3.3 (0.5-23.4)
9 to 12 months	5 (4.4%)	0 (0.0%)	NA
12 months or more	22 (19.3%)	0 (0.0%)	NA
Sex (adjusted for individual study participant, age and season)	114	27	
Female	50 (43.9%)	15 (55.6%)	ref
Male	64 (56.1%)	12 (44.4%)	0.5 (0.2-1.3)
Season (adjusted for individual study participant, age and sex)	114	27	
Dry (May to November)	71 (62.3%)	18 (66.7%)	ref
Wet (December to April)	43 (37.7%)	9 (33.3%)	0.2 (0.0-1.2)
**All variables adjusted for age, sex, season and individual study participant in GEE model**			
Campylobacter spp.	114	27	
No	102 (89.5%)	24 (88.9%)	ref
Yes	12 (10.5%)	3 (11.1%)	2.6 (0.5-12.4)
C. difficile	114	27	
No	102 (89.5%)	26 (96.3%)	ref
Yes	12 (10.5%)	1 (3.7%)	0.3 (0.0-2.2)
Plesiomonas	114	27	
No	113 (99.1%)	27 (100.0%)	
Yes	1 (0.9%)	0 (0.0%)	NA
Salmonella spp.	114	27	
No	110 (96.5%)	27 (100.0%)	
Yes	4 (3.5%)	0 (0.0%)	NA
Vibrio spp.	114	27	
No	111 (97.4%)	27 (100.0%)	
Yes	3 (2.6%)	0 (0.0%)	NA
Vibrio cholerae	114	27	
No	112 (98.2%)	27 (100.0%)	
Yes	2 (1.8%)	0 (0.0%)	NA
EAEC	114	27	
No	52 (45.6%)	12 (44.4%)	ref
Yes	62 (54.4%)	15 (55.6%)	1.6 (0.6-4.2)
EPEC	114	27	
No	73 (64.0%)	19 (70.4%)	ref
Yes	41 (36.0%)	8 (29.6%)	1.7 (0.6-5.3)
ETEC	114	27	
No	97 (85.1%)	22 (81.5%)	ref
Yes	17 (14.9%)	5 (18.5%)	2.0 (0.5-7.3)
STEC	114	27	
No	112 (98.2%)	27 (100.0%)	ref
Yes	2 (1.8%)	0 (0.0%)	1.5 (0.3-7.1)

E. coli O 157		114		27	
No	114 (100.0%)		27 (100.0%)		NA
Shigella /EIEC		114		27	
No	113 (99.1%)		26 (96.3%)		ref
Yes	1 (0.9%)		1 (3.7%)		3.0 (0.2-46.0)
Cryptosporidium		114		27	
No	111 (97.4%)		27 (100.0%)		ref
Yes	3 (2.6%)		0 (0.0%)		1.2 (0.3-4.0)
Cyclospora		114		27	
No	114 (100.0%)		27 (100.0%)		NA
Entamoeba		114		27	
No	114 (100.0%)		27 (100.0%)		NA
Giardia		114		27	
No	114 (100.0%)		26 (96.3%)		
Yes	0 (0.0%)		1 (3.7%)		NA
Adenovirus		114		27	
No	108 (94.7%)		27 (100.0%)		
Yes	6 (5.3%)		0 (0.0%)		NA
Astrovirus		114		27	
No	114 (100.0%)		27 (100.0%)		NA
Norovirus		114		27	
No	104 (91.2%)		25 (92.6%)		ref
Yes	10 (8.8%)		2 (7.4%)		1.5 (0.2-9.2)
Rotavirus		114		27	
No	108 (94.7%)		24 (88.9%)		ref
Yes	6 (5.3%)		3 (11.1%)		3.8 (0.4-35.5)
Sapovirus		114		27	
No	111 (97.4%)		26 (96.3%)		ref
Yes	3 (2.6%)		1 (3.7%)		1.5 (0.1-15.8)
