# Supplementary material for: A pilot study using hospital surveillance and a birth cohort to investigate enteric pathogens and malnutrition in children, Dili, Timor-Leste
Source: PLoS One. 2024 Feb 1;19(2):e0296774. doi: 10.1371/journal.pone.0296774 (PMC10833528; doi:10.1371/journal.pone.0296774)
Supplement: S3 Table — * Indicates statistical significance (p value <0.05). GEE aOR = adjusted odds ratio from generalised estimating equation model. 95% CI = 95% confidence interval. ref = reference variable. NA = odds ratio not calculated. (PDF) [file pone.0296774.s004.pdf]

**S3 Table. Adjusted univariate odds ratios using a generalised estimating equations model for risk factors associated with pathogen detection for infants in a birth cohort, Dili, Timor-Leste, 2019-2020.**

| | Pathogen detected (N=97) | No pathogen detected (N=44) | GEE aOR (95% CI) | |
|---|---|---|---|---|
| Age (adjusted for individual study participant and season) | 97 | 44 | | |
| Less than 3 months | 16 (16.5%) | 29 (65.9%) | ref | |
| 3 to 6 months | 27 (27.8%) | 9 (20.5%) | 2.8 (0.9-8.8) | |
| 6 to 9 months | 30 (30.9%) | 3 (6.8%) | 3.6 (0.4-32.1) | |
| 9 to 12 months | 4 (4.1%) | 0 (0.0%) | NA | |
| Over 12 months | 20 (20.6%) | 3 (6.8%) | 12.1 (3.1-47.0) | * |
| | | | | |
| Season (adjusted for individual study participant and age) | 97 | 44 | | |
| Dry (May to November) | 50 (51.5%) | 39 (88.6%) | ref | |
| Wet (December to April) | 47 (48.5%) | 5 (11.4%) | 5.1 (0.9-29.3) | |
| | | | | |
| **All variables adjusted for age, season and individual study participant in GEE model** | | | | |
| | | | | |
| Sex of child | 97 | 44 | | |
| Female | 43 (44.3%) | 22 (50.0%) | ref | |
| Male | 54 (55.7%) | 22 (50.0%) | 1.5 (0.7-3.5) | |
| | | | | |
| Household size | 97 | 44 | | |
| 5 or less | 14 (14.4%) | 9 (20.5%) | 0.6 (0.2-2.1) | |
| 6 to 10 | 61 (62.9%) | 24 (54.5%) | ref | |
| 11 to 15 | 20 (20.6%) | 10 (22.7%) | 1.0 (0.4-2.8) | |
| 16 or more | 2 (2.1%) | 1 (2.3%) | 0.4 (0.0-11.9) | |
| | | | | |
| Highest level of education by primary carer | 96 | 44 | | |
| Finished year 12 and/or further study | 60 (62.5%) | 30 (68.2%) | ref | |
| Did not complete schooling or finished before year 12 | 36 (37.5%) | 14 (31.8%) | 1.0 (0.4-2.5) | |

| | | | | |
|---|---|---|---|---|
| Primary feeding type of child | | 96 | 44 | |
| Breastfed | 31 (32.3%) | 26 (59.1%) | ref | |
| Bottlefed | 16 (16.7%) | 1 (2.3%) | 8.3 (1.1-62.7) | * |
| Combined breast and bottle | 22 (22.9%) | 14 (31.8%) | 1.6 (0.6-4.4) | |
| Combined plus solid | 27 (28.1%) | 3 (6.8%) | 0.9 (0.2-3.8) | |
| How is the bottle cleaned? | | 54 | 16 | |
| Cleaned | 41 (75.9%) | 15 (93.8%) | ref | |
| Rinse only | 13 (24.1%) | 1 (6.2%) | 0.9 (0.1-6.9) | |
| Where is food prepared? | | 96 | 44 | |
| Separate area (building or outdoors) | 47 (48.9%) | 19 (43.2%) | ref | |
| House | 49 (51.0%) | 25 (56.8%) | 0.7 (0.3-1.7) | |
| Is there a separate room in the house for kitchen? | | 49 | 25 | |
| Yes | 41 (83.7%) | 21 (84.0%) | ref | |
| No | 8 (16.3%) | 4 (16.0%) | 1.0 (0.2-4.6) | |
| How is garbage disposed? | | 97 | 44 | |
| Garbage bin or dumpster | 78 (80.4%) | 31 (70.5%) | ref | |
| Buried | 2 (2.1%) | 0 (0.0%) | NA | |
| Burned | 7 (7.2%) | 9 (20.5%) | 1.0 (0.3-3.3) | |
| Thrown away | 10 (10.3%) | 4 (9.1%) | 0.5 (0.1-3.4) | |
| What toilet facilities are available? | | 97 | 44 | |
| Latrine (pit or slab) | 94 (96.9%) | 43 (97.7%) | ref | |
| Flush toilet | 3 (3.1%) | 1 (2.3%) | 0.4 (0.0-5.2) | |
| What is the main water source for drinking water? | | 97 | 44 | |
| Municipal supply | 62 (63.9%) | 35 (79.5%) | ref | |
| Bore or ground | 16 (16.5%) | 6 (13.6%) | 2.4 (0.7-7.8) | |
| Bottled | 19 (19.6%) | 3 (6.8%) | 0.7 (0.1-3.1) | |

| | | | |
|---|---|---|---|
| What is the main source for washing water? | | 95 | 43 |
| Municipal | 74 (77.9%) | 35 (81.4%) | ref |
| Bore or ground | 20 (21.1%) | 7 (16.3%) | 2.1 (0.7-6.1) |
| Bottled | 1 (1.1%) | 1 (2.3%) | 0.1 (0.0-1.7) |
| Do you store water? | | 97 | 44 |
| Yes | 90 (92.8%) | 43 (97.7%) | ref |
| No | 7 (7.2%) | 1 (2.3%) | 0.7 (0.1-7.2) |
| Is the stored water covered? | | 67 | 41 |
| Always covered | 12 (17.9%) | 5 (12.2%) | ref |
| Mostly or sometimes covered | 55 (82.1%) | 36 (87.8%) | 0.3 (0.1-1.0) |
| Do you treat water before use? | | 97 | 44 |
| Yes | 70 (72.2%) | 40 (90.9%) | ref |
| No | 27 (27.8%) | 4 (9.1%) | 0.4 (0.1-3.0) |
| How many animals do you have? | | 72 | 34 |
| None | 1 (1.4%) | 1 (2.9%) | 0.5 (0.0-4.7) |
| Less than 5 | 45 (62.5%) | 17 (50.0%) | 1.5 (0.5-4.4) |
| 5 to 10 | 19 (26.4%) | 11 (32.4%) | ref |
| More than 10 | 7 (9.7%) | 5 (14.7%) | 0.6 (0.1-3.6) |
| How many farm animals (e.g., goats, chickens, cows) do you have? | | 72 | 34 |
| None | 15 (20.8%) | 6 (17.6%) | ref |
| Less than 5 | 34 (47.2%) | 16 (47.1%) | 1.0 (0.3-3.4) |
| 5 to 10 | 17 (23.6%) | 10 (29.4%) | 0.6 (0.2-2.3) |
| More than 10 | 6 (8.3%) | 2 (5.9%) | 0.6 (0.0-10.5) |
| How many pet animals (e.g., dogs, cats) do you have? | | 72 | 34 |
| None | 33 (45.8%) | 14 (41.2%) | ref |
| Less than 5 | 37 (51.4%) | 17 (50.0%) | 0.8 (0.3-2.1) |
| 5 to 10 | 2 (2.8%) | 3 (8.8%) | 0.5 (0.0-12.5) |

| | | | |
|---|---|---|---|
| Where are these animals located? | 68 | 33 | |
| Free outside | 17 (25.0%) | 11 (33.3%) | ref |
| Cage or pen | 11 (16.2%) | 0 (0.0%) | NA |
| Free inside | 29 (42.6%) | 15 (45.5%) | 1.5 (0.4-5.0) |
| Tied up | 11 (16.2%) | 7 (21.2%) | 1.7 (0.4-8.1) |
