# Supplementary material for: A pilot study using hospital surveillance and a birth cohort to investigate enteric pathogens and malnutrition in children, Dili, Timor-Leste
Source: PLoS One. 2024 Feb 1;19(2):e0296774. doi: 10.1371/journal.pone.0296774 (PMC10833528; doi:10.1371/journal.pone.0296774)
Supplement: S4 Table — * Indicates statistical significance (p value <0.05). GEE aOR = adjusted odds ratio from generalised estimating equation model. WHZ = weight-for-height z-score. 95% CI = 95% confidence interval. ref = reference variable. NA = odds ratio not calculated. (PDF) [file pone.0296774.s005.pdf]

S4 Table. Adjusted univariate odds ratios using a generalised estimating equations model for risk factors associated with moderate wasting for infants in a birth cohort, Dili, Timor-Leste, 2019-2020.

	Under -2 WHZ score (N=38)	Over -2 WHZ score (N=123)	GEE aOR (95% CI)	
Age (adjusted for individual study participant and season)	38	123		
Less than 3 months	9 (23.7%)	40 (32.5%)	ref	
3 to 6 months	10 (26.3%)	33 (26.8%)	2.8 (0.9-8.6)	
6 to 9 months	11 (28.9%)	25 (20.3%)	12.3 (1.7-87.6)	*
9 to 12 months	1 (2.6%)	6 (4.9%)	0.9 (0.1-8.1)	
Over 12 months	7 (18.4%)	19 (15.4%)	1.6 (0.5-5.1)	
Season (adjusted for individual study participant and age)	38	123		
Dry (May to November)	25 (65.8%)	77 (62.6%)	ref	
Wet (December to April)	13 (34.2%)	46 (37.4%)	0.2 (0.0-0.9)	*
**All variables adjusted for age, season and individual study participant in GEE model**				
Sex of child	38	123		
Female	19 (50.0%)	54 (43.9%)	ref	
Male	19 (50.0%)	69 (56.1%)	0.8 (0.4-1.7)	
Household size	38	123		
5 or less	2 (5.3%)	25 (20.3%)	0.3 (0.1-1.1)	
6 to 10	25 (65.8%)	71 (57.7%)	ref	
11 to 15	10 (26.3%)	25 (20.3%)	1.2 (0.5-2.9)	
16 or more	1 (2.6%)	2 (1.6%)	1.0 (0.1-9.7)	
Highest level of education by primary carer	38	121		
Finished year 12 and/or further study	28 (73.7%)	79 (65.3%)	ref	
Did not complete schooling or finished before year 12	10 (26.3%)	42 (34.7%)	0.7 (0.3-1.5)	

Primary feeding type of child	38	122	
Breastfed	12 (31.6%)	55 (45.1%)	ref
Bottlefed	4 (10.5%)	15 (12.3%)	1.2 (0.3-4.8)
Combined breast and bottle	11 (28.9%)	31 (25.4%)	1.6 (0.6-4.0)
Combined plus solid	11 (28.9%)	21 (17.2%)	5.9 (0.4-83.7)
How is the bottle cleaned?	20	59	
Cleaned	15 (75.0%)	44 (74.6%)	ref
Rinse only	5 (25.0%)	15 (25.4%)	2.9 (0.4-20.1)
Where is food prepared?	122	38	
Separate area (building or outdoors)	58 (47.5%)	20 (52.6%)	ref
House	64 (52.5%)	18 (47.4%)	0.7 (0.3-1.6)
Is there a separate room in the house for kitchen?	18	64	
Yes	14 (77.8%)	55 (85.9%)	ref
No	4 (22.2%)	9 (14.1%)	1.6 (0.4-5.9)
How is garbage disposed?	38	123	
Garbage bin or dumpster	29 (76.3%)	96 (78.0%)	ref
Buried	1 (2.6%)	2 (1.6%)	2.8 (0.3-31.1)
Burned	2 (5.3%)	15 (12.2%)	0.5 (0.1-3.0)
Thrown away	6 (15.8%)	10 (8.1%)	2.6 (0.7-10.4)
What toilet facilities are available?	38	123	
Latrine	38 (100.0%)	119 (96.7%)	
Flush	0 (0.0%)	4 (3.3%)	NA
What is the main water source for drinking water?	38	123	
Municipal supply	22 (57.9%)	84 (68.3%)	ref
Bore or ground	6 (15.8%)	20 (16.3%)	1.7 (0.5-5.1)
Bottled	10 (26.3%)	19 (15.4%)	2.5 (0.9-7.1)

What is the main source for washing water?		35	122
Municipal	29 (82.9%)	95 (77.9%)	ref
Bore or ground	5 (14.3%)	26 (21.3%)	0.8 (0.3-2.3)
Bottled	1 (2.9%)	1 (0.8%)	2.6 (0.1-46.7)
Do you store water?		38	123
Yes	35 (92.1%)	118 (95.9%)	ref
No	3 (7.9%)	5 (4.1%)	1.5 (0.3-7.4)
Is the stored water covered?		27	94
Always covered	4 (14.8%)	14 (14.9%)	ref
Mostly or sometimes covered	23 (85.2%)	80 (85.1%)	0.5 (0.1-1.8)
Do you treat water before use?		38	123
Yes	28 (73.7%)	96 (78.0%)	ref
No	10 (26.3%)	27 (22.0%)	1.2 (0.3-4.6)
How many animals do you have?		29	93
None	0 (0.0%)	2 (2.2%)	NA
Less than 5	20 (69.0%)	53 (57.0%)	0.8 (0.3-2.2)
5 to 10	8 (27.6%)	27 (29.0%)	ref
More than 10	1 (3.4%)	11 (11.8%)	0.3 (0.0-2.5)
How many farm animals (i.e., goats, chickens, cows) do you have?		29	93
None	6 (20.7%)	19 (20.4%)	ref
Less than 5	14 (48.3%)	44 (47.3%)	1.1 (0.4-3.2)
5 to 10	9 (31.0%)	22 (23.7%)	2.0 (0.6-6.9)
More than 10	0 (0.0%)	8 (8.6%)	NA
How many pet animals (i.e., dogs, cats) do you have?		29	93
None	11 (37.9%)	41 (44.1%)	ref
Less than 5	16 (55.2%)	48 (51.6%)	1.3 (0.5-3.4)
5 to 10	2 (6.9%)	4 (4.3%)	3.8 (0.6-23.5)

Where are these animals located?	28	89	
Free outside	11 (39.3%)	21 (23.6%)	ref
Cage/Pen	4 (14.3%)	10 (11.2%)	0.6 (0.1-2.5)
Free inside	10 (35.7%)	40 (44.9%)	0.6 (0.2-1.9)
Tied up	3 (10.7%)	18 (20.2%)	0.4 (0.1-1.9)
