# Supplementary material for: A pilot study using hospital surveillance and a birth cohort to investigate enteric pathogens and malnutrition in children, Dili, Timor-Leste
Source: PLoS One. 2024 Feb 1;19(2):e0296774. doi: 10.1371/journal.pone.0296774 (PMC10833528; doi:10.1371/journal.pone.0296774)
Supplement: S5 Table — (PDF) [file pone.0296774.s006.pdf]

S5 Table. Weight-for-height, height-for-age, and weight-for-age, and MUAC-for-age for hospital cohort children at admission, Dili, Timor-Leste, 2019-2020.

	Weight-for-height z-score (median, IQR)	Wasted *n* (%, 95%CI)§	Height-for-age z-score (median, IQR)	Stunted *n* (%, 95%CI)§	Weight-for-age z-score (median, IQR)	Underweight *n* (%, 95%CI)§	MUAC-for-age z-score (median, IQR)	Acutely malnourished *n* (%, 95%CI)§	Number of children (% female)	Age in months (mean, ±*SD*)
**All**	-3.7 (-4.7 to -2.9)	135/153* (88.2, 81.8 to 92.7)	-2.5 (-3.5 to -1.1)	97/156* (62.2, 54.0 to 69.7)	-3.8 (-4.6 to -3.1)	141/157* (89.8, 83.7 to 93.9)	-3.5 (-4.3 to -2.9)	130/144* (90.3, 83.9 to 94.4)	157/159* (52.9)	17.7 (11.4)
**SAM**	-3.9 (-4.7 to -3.0)	124/134* (92.5, 86.4 to 96.2)	-2.6 (-3.6 to -1.4)	88/137* (64.2, 55.5 to 72.1)	-3.9 (-4.8 to -3.2)	128/138* (92.8, 86.7 to 96.3)	-3.7 (-4.3 to -3.2)	120/128* (93.8, 87.7 to 97.1)	138/140* (54.3)	17.5 (10.8)
**Severe diarrhoea**	-2.6 (-4.2 to -1.1)	11/19 (57.9, 34.0 to 78.9)	-1.4 (-2.8 to -0.5)	9/19 (47.4, 25.2 to 70.5)	-3.0 (-3.7 to -1.4)	13/19 (68.4, 43.5 to 86.4)	-2.3 (-2.9 to -1.0)	10/16* (62.5, 35.9 to 83.7)	19 (42.1)	18.9 (15.6)

* calculations do not include all 159 children due to missing data or having measurements below the lower calculation thresholds. SAM = severe acute malnutrition. IQR = interquartile range. *n* = number of cases. 95% CI = 95% confidence interval. *SD* = standard deviation. MUAC = middle-upper arm circumference. § wasted, stunted, underweight, and acutely malnourished includes moderate (≤-2) and severe (≤-3) z-scores.
