# Supplementary material for: A pilot study using hospital surveillance and a birth cohort to investigate enteric pathogens and malnutrition in children, Dili, Timor-Leste
Source: PLoS One. 2024 Feb 1;19(2):e0296774. doi: 10.1371/journal.pone.0296774 (PMC10833528; doi:10.1371/journal.pone.0296774)
Supplement: S6 Table — (PDF) [file pone.0296774.s007.pdf]

S6 Table. Mean age in months at admission by diagnosis and type of enteric pathogen detected for hospital-based surveillance cases, Dili, Timor-Leste, 2019–2020.

**Enteric pathogen type**	**All hospitalised cases (mean age in months, ±*SD*)**	**SAM cases (mean age in months, ±*SD*)**	**Severe diarrhoea (mean age in months, ±*SD*)**
**Bacteria***	17.8 (11.2)	17.5 (10.6)	19.8 (14.8)
**Parasites†^**	19.3 (11.7)	18.8 (11.4)	25.0 (14.9)
**Viruses‡**	13.8 (8.1)	14.0 (8.5)	12.5 (5.1)

SD = standard deviation. SAM = severe acute malnutrition. *Bacteria include *Campylobacter* spp., *Clostridioides difficile, Plesiomonas shigelloides, Salmonella* spp., *Vibrio* spp., diarrhoeagenic *Escherichia coli,* and *Shigella.* †Parasites include *Cryptosporidium* spp., *Giardia lamblia*, *Cyclospora cayetanensis*, and *Entamoeba histolytica*. ‡Viruses include adenovirus, astrovirus, norovirus, rotavirus, and sapovirus. ^ indicates p value < 0.05 between SAM and severe diarrhoea diagnosis groups.
