# Supplementary material for: A pilot study using hospital surveillance and a birth cohort to investigate enteric pathogens and malnutrition in children, Dili, Timor-Leste
Source: PLoS One. 2024 Feb 1;19(2):e0296774. doi: 10.1371/journal.pone.0296774 (PMC10833528; doi:10.1371/journal.pone.0296774)
Supplement: S7 Table — * Indicates statistical significance (p value <0.05). aOR = adjusted odds ratio. 95% CI = 95% confidence interval. ref = reference variable. NA = odds ratio not calculated. (PDF) [file pone.0296774.s008.pdf]

S7 Table. Adjusted univariate odds ratios using a generalised linear model for differences in pathogens detected between diarrhoeal and non-diarrhoea stool samples for children from a hospital-based surveillance cohort in Dili, Timor-Leste, 2019-2020.

	Solid or semi-solid stool (N=136)	Watery stool with or without blood (N=11)	aOR (95% CI)	
Age (adjusted for sex and season)	136	11		
0 to 12 months	52 (38.2%)	3 (27.3%)	ref	
1 to 2 years	56 (41.2%)	5 (45.5%)	1.6 (0.4-8.5)	
2 to 3 years	14 (10.3%)	2 (18.2%)	2.6 (0.3-18.0)	
3 to 4 years	11 (8.1%)	1 (9.1%)	1.8 (0.1-16.0)	
4 to 5 years	3 (2.2%)	0 (0.0%)	NA	
Sex (adjusted for age and season)	136	11		
Female	71 (52.2%)	7 (63.6%)	ref	
Male	65 (47.8%)	4 (36.4%)	0.6 (0.2-2.2)	
Season (adjusted for age and sex)	136	11		
Dry (May to November)	40 (29.4%)	3 (27.3%)	ref	
Wet (December to April)	96 (70.6%)	8 (72.7%)	0.9 (0.2-4.5)	
**All variables adjusted for age, sex, season in generalised linear model**				
Campylobacter spp.	136	11		
No	104 (76.5%)	7 (63.6%)	ref	
Yes	32 (23.5%)	4 (36.4%)	1.8 (0.4-6.8)	
C. difficile	136	11		
No	131 (96.3%)	11 (100.0%)		
Yes	5 (3.7%)	0 (0.0%)	NA	
Plesiomonas	136	11		
No	135 (99.3%)	11 (100.0%)		
Yes	1 (0.7%)	0 (0.0%)	NA	
Salmonella spp.	136	11		
No	133 (97.8%)	9 (81.8%)	ref	
Yes	3 (2.2%)	2 (18.2%)	17.2 (1.6-201.3)	*
Vibrio spp.	136	11		
No	131 (96.3%)	11 (100.0%)		
Yes	5 (3.7%)	0 (0.0%)	NA	
Vibrio cholerae	136	11		
No	133 (97.8%)	11 (100.0%)		
Yes	3 (2.2%)	0 (0.0%)	NA	
EAEC	136	11		
No	49 (36.0%)	5 (45.5%)	ref	
Yes	87 (64.0%)	6 (54.5%)	0.6 (0.2-2.3)	
EPEC	136	11		
No	63 (46.3%)	4 (36.4%)	ref	
Yes	73 (53.7%)	7 (63.6%)	1.5 (0.4-6.8)	
ETEC	136	11		
No	94 (69.1%)	8 (72.7%)	ref	
Yes	42 (30.9%)	3 (27.3%)	0.7 (0.1-2.9)	
STEC	136	11		
No	132 (97.1%)	11 (100.0%)		
Yes	4 (2.9%)	0 (0.0%)	NA	
E. coli O157	136	11		
No	134 (98.5%)	11 (100.0%)		
Yes	2 (1.5%)	0 (0.0%)	NA	

Shigella /EIEC		136	11	
No	111 (81.6%)	6 (54.5%)	ref	
Yes	25 (18.4%)	5 (45.5%)	3.8 (1.0-14.4)	*
Cryptosporidium		136	11	
No	121 (89.0%)	8 (72.7%)	ref	
Yes	15 (11.0%)	3 (27.3%)	3.0 (0.6-12.3)	
Cyclospora		136	11	
No	125 (91.9%)	10 (90.9%)	ref	
Yes	11 (8.1%)	1 (9.1%)	0.9 (0.0-6.0)	
Entamoeba		136	11	
No	135 (99.3%)	11 (100.0%)		
Yes	1 (0.7%)	0 (0.0%)	NA	
Giardia		136	11	
No	107 (78.7%)	7 (63.6%)	ref	
Yes	29 (21.3%)	4 (36.4%)	2.2 (0.5-8.7)	
Adenovirus		136	11	
No	133 (97.8%)	10 (90.9%)	ref	
Yes	3 (2.2%)	1 (9.1%)	5.5 (0.2-58.7)	
Astrovirus		136	11	
No	129 (94.9%)	11 (100.0%)		
Yes	7 (5.1%)	0 (0.0%)	NA	
Norovirus		136	11	
No	124 (91.2%)	10 (90.9%)	ref	
Yes	12 (8.8%)	1 (9.1%)	1.0 (0.1-6.0)	
Rotavirus		136	11	
No	130 (95.6%)	10 (90.9%)	ref	
Yes	6 (4.4%)	1 (9.1%)	2.7 (0.1-24.7)	
Sapovirus		136	11	
No	123 (90.4%)	10 (90.9%)	ref	
Yes	13 (9.6%)	1 (9.1%)	0.9 (0.0-6.0)	
