# Supplementary material for: A pilot study using hospital surveillance and a birth cohort to investigate enteric pathogens and malnutrition in children, Dili, Timor-Leste
Source: PLoS One. 2024 Feb 1;19(2):e0296774. doi: 10.1371/journal.pone.0296774 (PMC10833528; doi:10.1371/journal.pone.0296774)

S1 Fig. Total pathogen detections for a birth cohort and hospital surveillance cohort, stratified by age group, in infants and children in Dili, Timor-Leste, 2019–2020.

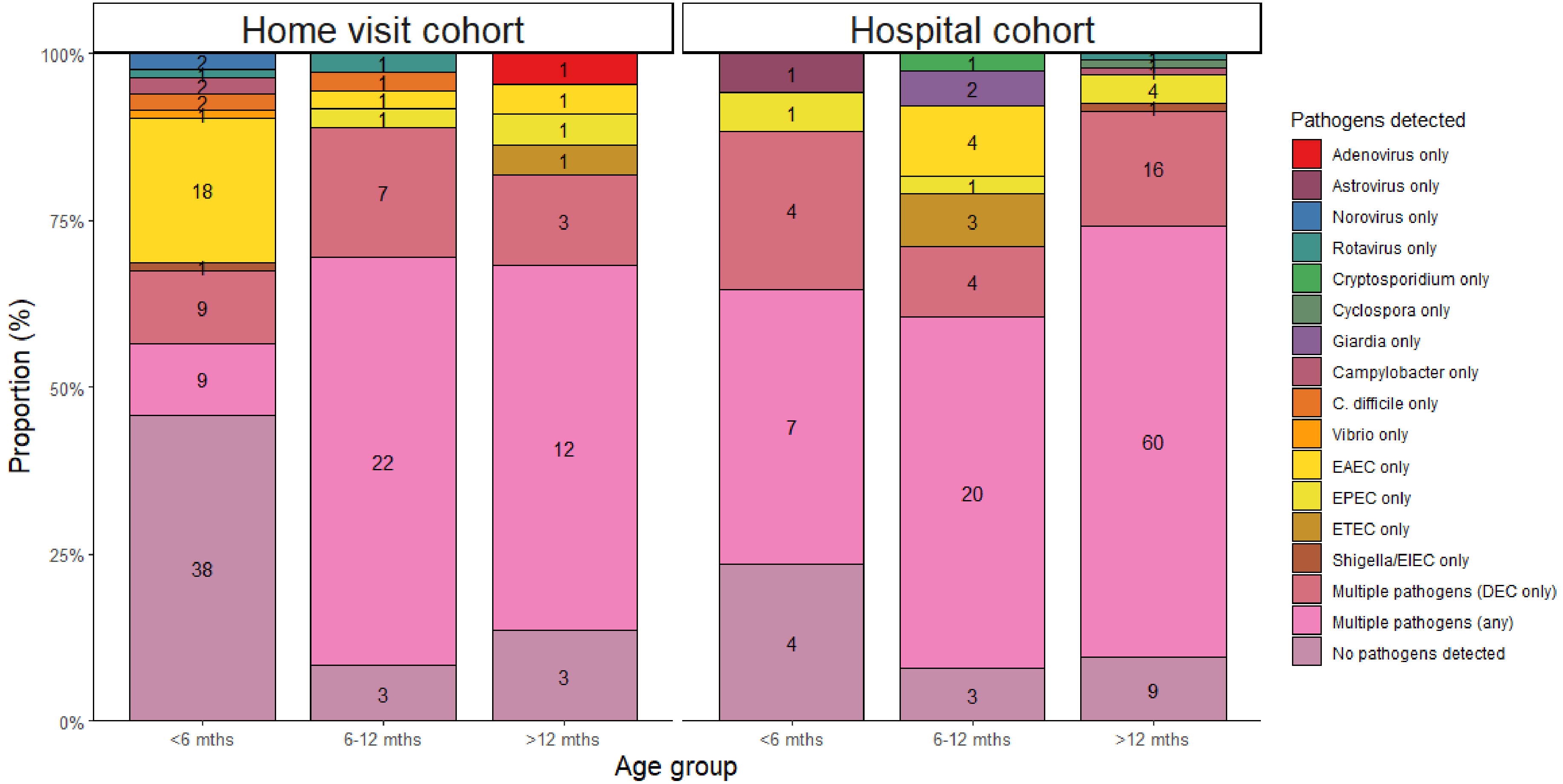

Supplement: S1 Fig — (PDF) [file pone.0296774.s009.pdf]
