# Supplementary material for: A pilot study using hospital surveillance and a birth cohort to investigate enteric pathogens and malnutrition in children, Dili, Timor-Leste
Source: PLoS One. 2024 Feb 1;19(2):e0296774. doi: 10.1371/journal.pone.0296774 (PMC10833528; doi:10.1371/journal.pone.0296774)
Supplement: S1 File — (PDF) [file pone.0296774.s010.pdf]

S1 File. Questionnaire administered to families during birth cohort home visits in Dili, Timor-Leste, 2019–2020.

FAMILY QUESTIONNAIRE (PARENT/CAREGIVER)

Stunting and gastrointestinal infections in infants in Timor-Leste: A pilot study

Attached is a paper version of the electronic questionnaire that will be administered by researchers to the parent or guardian of the participating infant at each of the four home visits.

The questionnaire will be created using the secure Research Electronic Data Capture (REDCap) software https://projectredcap.org/software/.

The researcher will ask all the questions and enter the data directly into the questionnaire on a laptop or tablet, on return to the office they will upload it to the database and the data will be erased from the laptop/tablet.

Most of the questions are copied verbatim from the Timor-Leste Demographic and Health Survey 2016. These questions were tested and validated prior to the survey. Other questions are adapted from a previously approved study: *Should integrated deworming and WASH programs for STH control be delivered in schools or the community? – a pilot study in preparation of a cluster randomized trial* GECQ Reference Number: GECQ/EP/2015/025

FAMILY QUESTIONNAIRE (PARENT/CAREGIVER)

Stunting and gastrointestinal infections in infants in Timor-Leste: A pilot study

Address:

Date : ☐☐ / ☐☐ / 2 0 ☐☐ Interviewer initials: ☐☐

Consent checklist

Has written consent been obtained? Y ☐ N ☐ *Only proceed if yes*

A. Baby details

1. Participant ID: ☐☐☐☐☐☐☐☐

2. Name: ☐

3. Gender: M ☐ F ☐

4. Date of Birth (DD/MM/YYYY): ______________________

5. Name of caregiver: ☐

6. Relationship with participant ☐

7. How many people of the following age groups live in your household?

<5 years	
5-17 years	
18-65 years	
>65 years	
Total	

B. Family Health

9. To your knowledge, has your baby taken antibiotic medicine since birth/our last visit? Y ☐ N ☐ Refused ☐

10. In the last 24 hours, how many bowel motions has your baby passed? ☐

11. In the past 24 hours, describe your baby's bowel motions:
(Tick ONLY ONE correct response)
- Normal ☐
- Loose and/or watery ☐
- Loose and/or watery with blood ☐

12. In the last two weeks has your baby had loose stools 3 or more times in one day? Y ☐ N ☐

If yes, was there any visible blood in his/her stools? Y ☐ N ☐

13. In the last 24 hours have you, or anyone else living with you (other than the baby), had loose stools 3 or more times? Y ☐ N ☐

If yes, was there any visible blood in his/her stools? Y ☐ N ☐

C. Food preparation

14. How is your baby fed?

Tick (√) all items mentioned WITHOUT reading out options:

Breast-fed			
Bottle-fed		Specify:	
Solid foods		Specify:	
Other		Specify:	
Don’t know			
Refused			

15. If bottle-fed, how do you clean the bottle between feeds?

Tick (√) all items mentioned WITHOUT reading out options:

Rinse with water			
Wash with soap			
Boil			
Other		Specify:	
Don’t know			
Refused			

16. Is the cooking for the family usually done in the house, in a separate building, or outdoors?

Tick (√) one item only:

In the house		Go to 17	
In a separate building			
Outdoors under cover			
Outdoors			
Other		Specify:	
Don’t know			
Refused			

17. Do you have a separate room which is used as a kitchen? Y ☐ N ☐ Refused ☐

D. Sanitation data

18. How do you usually dispose of your household garbage?

Tick (√) one item only:

Burned			
Buried			
Thrown away			
Garbage bin/dumpster and picked up			
Recycled			
Other		Specify:	
Don’t know			
Refused			

19. What kind of toilet facility do members of your household usually use?

Tick off (√) toilet type, using picture sheet of different toilet types as a guide:

Flush to septic tank			
Flush to pit latrine			
Flush to somewhere else		Specify:	
Flush don’t know where			
Ventilated improved pit latrine			

Pit latrine with slab			
Pit latrine without slab/open pit			
Composting toilet			
Bucket toilet			
Hanging toilet or latrine			
No facility/bush/field			
Other		Specify:	
Don't know			
Refused			

20. What are the sources of drinking water for your household?

Read all options and tick (√) all items mentioned. Show the picture sheet of different water sources. Observe sources if possible:

Piped water into dwelling (inside house)			
Piped water to yard/plot - this house only			
Piped water shared with other houses (to yard/plot or to communal place)			
Public tap/standpipe			
Tubewell or borehole or protected dug well			
Unprotected dug well			
Protected spring			
Unprotected spring			
Rainwater			
Tanker truck			
Surface water			
Bottled water			
Other		Specify:	
Don't know			
Refused			

21. What are the sources of water used by your household for other purposes such as cooking and handwashing?

Read all options and tick (√) all items mentioned. Show the picture sheet of different water sources. Observe source if possible:

Piped water into dwelling (inside house)			
Piped water to yard/plot - this house only			
Piped water shared with other houses (to yard/plot or to communal place)			
Public tap/standpipe			
Tubewell or borehole or protected dug well			
Unprotected dug well			
Protected spring			
Unprotected spring			
Rainwater			
Tanker truck			
Surface water			
Bottled water			
Other		Specify:	
Don't know			
Refused			

22. Where is the main water source located?

CHOOSE ONLY ONE OPTION with a tick (√):

In your house	
In your yard or plot	

Other		Specify:	
Don’t know			
Refused			

23. Do you store water from this main source in the household? Y ☐ N ☐ *If no, skip to question 25.*

24. If yes, what type of container(s) do you use to store water?

Tick all items mentioned or demonstrated:

Jerry-can		Covered?	All		Some		None			
Balde/basin		Covered?	All		Some		None			
Ceramic pot		Covered?	All		Some		None			
Other		Covered?	All		Some		None		Specify:	
Don’t know										
Refused										

25. Do you do anything to your water to make it safer? Y ☐ N ☐ *If no, skip to question 28.*

26. If yes, which purposes would you treat the water for?

Read all options and tick (√) all items mentioned:

Drinking			
Cooking			
Dishwashing			
Hand/body washing			
Other		Specify:	
Don’t know			
Refused			

27. What do you treat it with? *Read all options and tick (√) all items mentioned:*

Household bleach			
Boil			
Strain through cloth			
Filter			
Other		Specify:	
Don’t know			
Refused			

28. What is your highest level of education? *Tick only one option.*

Never went to school	
Not finished primary school (6 years)	
Completed primary school	
Not finished pre-secundario (9 years)	
Completed pre-secundario (9 years)	
Not finished secundario (12 years)	
Completed secundario (12 years)	
Completed professional training	
Not finished university	
Completed university	
Don’t know	
Refused	

29. What is your current employment status?

Employed/has job		→ Specify MAIN job:		
Doing housework				
Student				

Retired	
Long-term disabled	
Unemployed	
Don't know	
Refused	

30. How much income did your household receive over the last year?

Less than USD $365	
USD $365-730	
USD $730-1460	
More than USD $1460	
Don't know	
Refused	

31. Does your household keep any of the following animals?

Dogs? Y		N		If yes, how many?		Where are they kept?	
Pigs? Y		N		If yes, how many?		Where are they kept?	
Chickens? Y		N		If yes, how many?		Where are they kept?	
Cows/buffalo? Y		N		If yes, how many?		Where are they kept?	
Other animals? Y		N		If yes, which and how many?		Where are they kept?	
Don't know							
Refused							

32. Observe presence of animals moving freely inside or around the house. Select all that apply.

Animals in pens/cages	
Animals moving freely outside	
Animals moving freely inside	

This completes the questionnaire. We are grateful for your participation - thank you.
