# Supplementary figures and images for: A pilot study using hospital surveillance and a birth cohort to investigate enteric pathogens and malnutrition in children, Dili, Timor-Leste
Source: PLoS One. 2024 Feb 1;19(2):e0296774. doi: 10.1371/journal.pone.0296774 (PMC10833528; doi:10.1371/journal.pone.0296774)

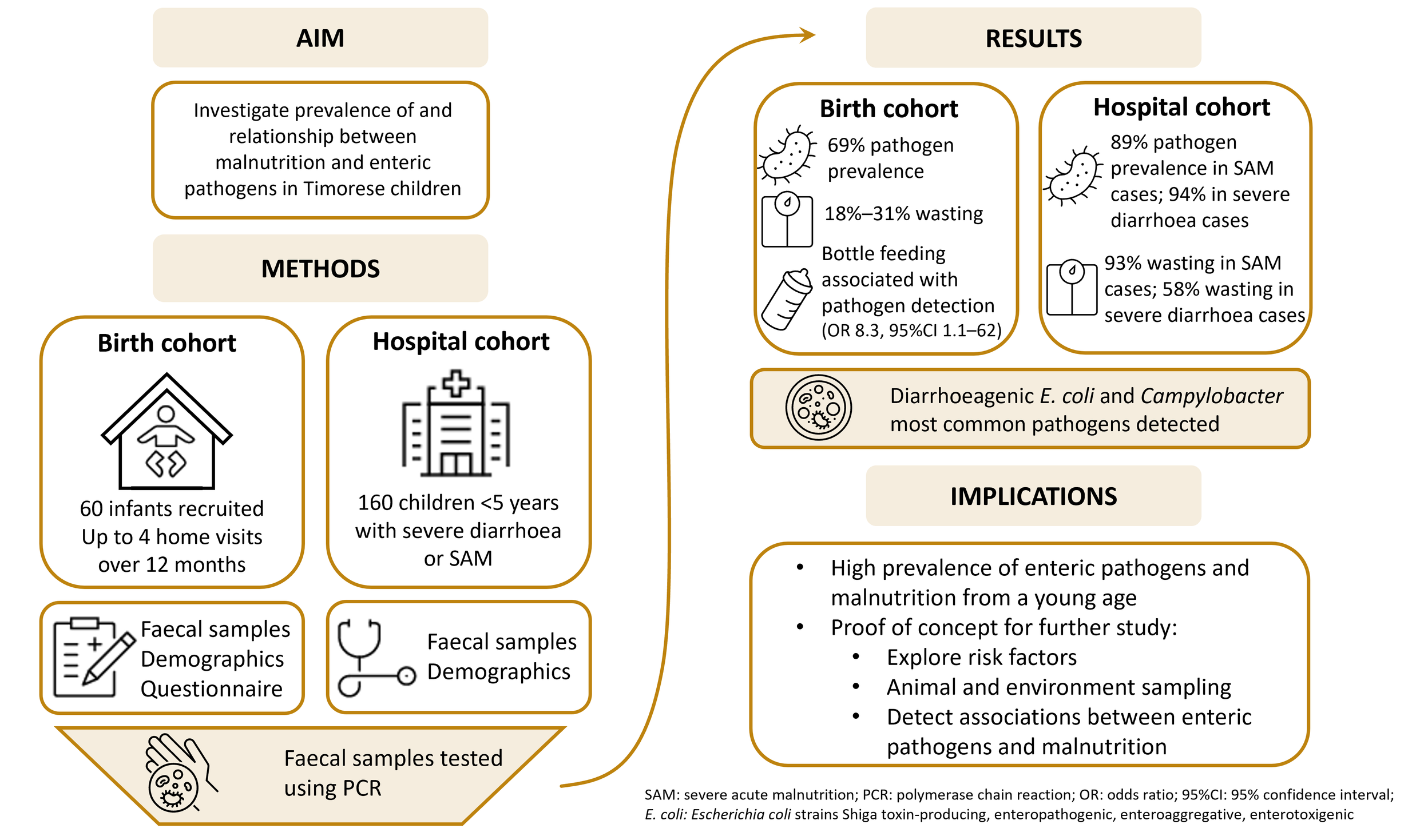

Supplement: S1 Graphical abstract — (TIF) [file pone.0296774.s011.tif]
