# Supplementary material for: A pilot study using hospital surveillance and a birth cohort to investigate enteric pathogens and malnutrition in children, Dili, Timor-Leste
Source: PLoS One. 2024 Feb 1;19(2):e0296774. doi: 10.1371/journal.pone.0296774 (PMC10833528; doi:10.1371/journal.pone.0296774)
Supplement: Attachment — Submitted filename: ReviewerResponse.pdf [file pone.0296774.s012.pdf]

09/08/2023

Dear Dr António Machado and reviewers,

We thank you and the reviewers for the time to review our manuscript PONE-D-23-14515 entitled "A pilot study using hospital surveillance and a birth cohort to investigate enteric pathogens and malnutrition in children, Dili, Timor-Leste." We have reviewed and addressed the comments and suggestions from the reviewers in a point-by-point response below. Reviewer comments are in black text and our responses and changes within the manuscript are in blue text. Line numbers refer to the 'simple markup' track changes version of the document.

Reviewer 1

The authors present their work regarding enteric pathogens and malnutrition in Timor-Leste and expected to find out the risk factors associated with them. However; the study presented a lot of data in every possible manner with no focus on the actual focus on the actual objective of the study.

Since its a pilot study, it should also collect some significant number of fecal samples from community settings. The number of sample size is not adequate to come to a conclusion.

We collected 141 faecal samples from children within the community (birth cohort). We acknowledge that there is a small sample size due to the pilot nature of the study, and that due to the different characteristics of the study groups, we were unable to combine these samples with the hospitalised children's samples to create a larger sample size. We have specifically pointed out this limitation where relevant in our manuscript (results and discussion). We still believe that our findings provide a suitable overview of the prevalence of pathogens and malnutrition to inform a further study on these factors in children in Dili.

Line 267 – 271: While we did not detect a significant relationship between WHZ and pathogens detected for visits one, two, and four (linear model *p* value >0.05), we did detect a significant negative trend in visit three (*p* value <0.01). However, we note that the small sample size and wide confidence intervals limit the interpretation of these results (Fig 2).

Line 352 – 354: This is most likely due to the small sample size of our study but could also be related to multiple pathogen interactions or additional nutritional or environmental factors.

Q 1. a BioFire® PCR assay was performed to test all faecal samples-How PCR was performed, since it is a multiplex technique, how many primers and samples were loaded at a time?
No methodology for PCR was mentioned, no images and results for PCR was shared, please share
Was it performed in-house or outsourced?

We have provided further details on the PCR methods in our methods section to address these queries. PCR analysis was performed in-house at the Hospital Laboratory in Dili. We have referenced the BioFire protocol document which provides in-depth description of methods including primer use. We used 30-test kits. Further, we have provided PCR detection results in Tables 1 and 2 and have created Fig 1 to illustrate the sample and testing process for the birth cohort.

Line 177 – 196: Laboratory staff processed samples from the birth cohort and hospital surveillance cohort using the same method. The NHL received faecal samples and performed visual inspections of the stool, routine microscopy including motility, and Gram stain morphology. In line with the standardised protocol, samples were stored either at room temperature (15-25°C) or refrigerated (2-

8°C) for up to four days [1]. Laboratory staff transferred approximately 1g of stool to a screw-cap tube filled with 15ml of Cary Blair liquid medium (Remel, Lenexa, KS, USA) which was shaken until homogenous [2]. The NHL used the BioFire® Gastrointestinal Panel (GI) Kit for in-house PCR testing on all faecal samples from birth cohort and hospitalised children against 22 gastrointestinal pathogens, namely *Campylobacter* (*C. jejuni*/*C. coli*/*C. upsaliensis*), *Clostridioides* (*Clostridium*) *difficile* (toxin A/B), *Plesiomonas shigelloides*, *Salmonella*, *Yersinia enterocolitica*, *Vibrio* (*V. parahaemolyticus*/*V. vulnificus*/*V. cholerae*), Enteroaggregative *E. coli* (EAEC), Enteropathogenic *E. coli* (EPEC), ETEC lt/st, Shiga-like toxin-producing *E. coli* (STEC) stx1/stx2, *E. coli* O157, *Shigella*/Enteroinvasive *E. coli* (EIEC), Adenovirus F40/41, Astrovirus, Norovirus GI/GII, Rotavirus A, Sapovirus (I, II, IV, and V), *Cryptosporidium*, *Cyclospora cayetanensis, Entamoeba histolytica*, and *Giardia lamblia*. Samples were prepared using a 30-test kit (RFIT-ASY-0116, bioMérieux Australia PTY LTD, AU) according to manufacturer's instructions [1]. We considered samples with a positive PCR result for two or more of the screened pathogens to have multiple pathogens detected. Samples positive by BioFire® PCR for selected enteric bacteria (*Campylobacter, Salmonella* and *Shigella*/EIEC) were reflex cultured on appropriate selective media and an animal health team returned to the household to collect animal samples.

Line 254–256: **Fig 1. Birth cohort recruitment and polymerase chain reaction (PCR) results in 60 infants, Dili, Timor-Leste, 2019–2020.** *See Table 1 for details on pathogens detected; +ve = positive, -ve = negative, LFTU = loss to follow up.

Q 2. When you mention that 63.9% isolates were positive for multiple pathogens (how many numbers you consider to call it multiple pathogens}?

We defined multiple pathogen infections as two or more screened pathogens detected in a sample at each visit. We have provided a definition of this in our methods section.

Line 192 – 193: We considered samples with a positive PCR result for two or more of the screened pathogens to have multiple pathogens detected.

3. Please write no of total samples in tables above

We have already included the total number of samples in Tables 1 and 2 within our manuscript. These are included in the final table rows prior to the table legend and are in bolded font.

Lines 272 (Table 1) and 322 (Table 2)

4. Methodology is confusing and appears to be non-repetitive

We have amended specific issues with our methodology and have reorganised sections to be clearer to understand. We have added additional details on study recruitment, specimen collection, pathogen detection techniques using PCR, and provided specific details on analysis strategies depending on the dataset used. These additional details should make the methods more reproducible.

Line 124 – 238

5. Results are misleading as different numbers are mentioned at different places

We have carefully reviewed our results to check for consistency in reported numbers. Further, we have changed, removed, or moved to the supplementary materials any confusing or non-essential statistics. This included changing reported Z scores to proportion of cases with wasting, stunting, or underweight to provide more context, and removing the section where diarrhoeal stools were compared as a combined analysis between cohorts. Where different numbers were reported for the

same cohort (e.g., 19 severe diarrhoea cases under hospital surveillance with 16 stool samples submitted), the differences in these numbers are clearly explained.

Line 300 – 302: Hospital doctors diagnosed 140 (88.1%, 95% CI 81.7–92.5%) children with SAM (132 with stool samples submitted to the NHL) and 19 (11.9%, 95% CI 7.5–18.3%) with severe diarrhoeal disease (13 with DWSD and six with dysentery, 16 with stool samples submitted to the NHL).

6. Risk factors and their associations with enteric pathogens and malnutrition in Timor-Leste is not explored as mentioned in the conclusion.

We explored risk factors in the birth cohort with results presented in-text and in supplementary tables 3 and 4. We found that when adjusted for age, sex, season and repeated observations, exclusive bottle feeding was associated with increased odds of pathogen detection. No other risk factors were significant for pathogen detection or wasting analyses.

Line 286 – 293: Odds of pathogen detection increased with age, peaking in those aged over 12 months (OR 12.1, 95% CI 3.1-47.0) compared with children aged <3 months (S3 Table). We controlled for age, season, and repeated observations of individual participants in analysis of all additional risk factors. We found that exclusive bottle feeding was associated with increased odds of enteric pathogen detection (aOR 8.3, 95% CI 1.1-62.7) compared with breastfeeding. No other factors, including environmental and animal-related variables, were statistically significant. For the outcome of wasting, controlled for age, season, and repeated observations, we did not find any significant associations between risk factors (S4 Table).

7. Massive English language writing skills are required as it is full of grammatical and typo errors.

We have closely reviewed our manuscript for any grammatical or spelling errors. We are using Australian (British) English throughout the manuscript, except where words are in Tetum or Latin (pathogen names), or for proper names (e.g., World Health Organization).

8. Methodology and results showed few lines for PCR but the discussion is just on PCR, which was not the main aim of the study.

We have included more detailed information in our methods and results on the PCR technology used. As enteric pathogen prevalence was a focus of our paper and our strongest finding and that this is a novel area of research in Timor-Leste, we have maintained a strong focus in our discussion on the implications of enteric pathogens in childhood malnutrition.

Line 177 – 196: Laboratory staff processed samples from the birth cohort and hospital surveillance cohort using the same method. The NHL received faecal samples and performed visual inspections of the stool, routine microscopy including motility, and Gram stain morphology. In line with the standardised protocol, samples were stored either at room temperature (15-25°C) or refrigerated (2-8°C) for up to four days [1]. Laboratory staff transferred approximately 1g of stool to a screw-cap tube filled with 15ml of Cary Blair liquid medium (Remel, Lenexa, KS, USA) which was shaken until homogenous [2]. The NHL used the BioFire® Gastrointestinal Panel (GI) Kit for in-house PCR testing on all faecal samples from birth cohort and hospitalised children against 22 gastrointestinal pathogens, namely *Campylobacter* (*C. jejuni*/*C. coli*/*C. upsaliensis*), *Clostridioides* (*Clostridium*) *difficile* (toxin A/B), *Plesiomonas shigelloides*, *Salmonella*, *Yersinia enterocolitica*, *Vibrio* (*V. parahaemolyticus*/*V. vulnificus*/*V. cholerae*), Enteroaggregative *E. coli* (EAEC), Enteropathogenic *E. coli* (EPEC), ETEC lt/st, Shiga-like toxin-producing *E. coli* (STEC) stx1/stx2, *E. coli* O157, *Shigella*/Enteroinvasive *E. coli* (EIEC), Adenovirus F40/41, Astrovirus, Norovirus GI/GII, Rotavirus A, Sapovirus (I, II, IV, and V), *Cryptosporidium*, *Cyclospora cayetanensis, Entamoeba histolytica*, and *Giardia lamblia*. Samples were prepared using a 30-test kit (RFIT-ASY-0116, bioMérieux Australia PTY

LTD, AU) according to manufacturer's instructions [1]. We considered samples with a positive PCR result for two or more of the screened pathogens to have multiple pathogens detected. Samples positive by BioFire® PCR for selected enteric bacteria (*Campylobacter, Salmonella* and *Shigella*/EIEC) were reflex cultured on appropriate selective media and an animal health team returned to the household to collect animal samples.

9. One Health collaborative approach is a good idea to work on this study but it was merely pointed as a suggestion

We have re-included our methods and results on animal sampling. We conducted limited animal sampling due to an African Swine Fever outbreak that occurred in the middle of our study. Where samples were collected, the animal health team successfully cultured three *Campylobacter* isolates. With this context within our manuscript, our suggestions for further collaboration are now supported.

Line 193 – 196: Samples positive by BioFire® PCR for selected enteric bacteria (Campylobacter, Salmonella and Shigella/EIEC) were reflex cultured on appropriate selective media and an animal health team returned to the household to collect animal samples.

Line 283 – 285: The animal health team collected and cultured three animal samples from PCR-positive *Campylobacter* human samples from households with chickens and/or pigs. All three samples were culture-positive for *Campylobacter*.

10. The study started with a lot of promises and data to be explored but concluded randomly with no definite outcome and conclusion.

We have rewritten our conclusion in line with suggestions from this and other reviewers to provide further relevant outcomes and summaries in line with our data rather than in the general literature.

Line 445 – 454: We detected high prevalence of enteric pathogens, particularly the bacterial pathogens DEC and *Campylobacter* spp., and a high proportion of wasting from a young age in children in Dili, Timor-Leste. While we faced local challenges, we have identified strategies for mitigating these issues in the future and have produced practical information to guide the development of the next phase of this study. We suggest further exploration of risk factors in a high-powered study with a focus on feeding practices (e.g., exclusive bottle feeding) and the risk of enteric pathogen infection. Further, we recommend greater sampling from animal and environmental sources to identify potential transmission pathways. This would utilise a One Health collaborative approach between hospital and human health laboratory professionals and animal health experts including veterinarians and laboratory specialists to enhance local skills and capacity.

Reviewer 2

This pilot study aimed to understand the landscape of malnutrition and enteric pathogens among children in Dili, Timor-Leste. The authors rationalize, and I agree, that this study has the potential to provide important framework for the development of larger studies aimed at understanding risk factors and outcomes of malnutrition and enteric pathogens among children in this community. The authors have clearly worked hard on this study, and it does offer helpful information about enteropathogens in a region of the world that is a bit of data blindspot. However, to have the utility that the authors hope for, this manuscript needs some major revisions, in addition to the changes made in response to the previous round of reviews.

Major Revisions

Introduction: In the paragraph beginning at line 102, the authors introduce the 3 aims of this study. Aim 1 states that they planned to “estimate the proportion of children from a birth cohort who were malnourished or stunted at any time during the first 12 months of life”. However, I do not see any results in the manuscript that address this aim. The authors share the average z-scores among children in the cohort, but do not present a proportion of birth cohort children with malnutrition or wasting in the 12-month period.

We have calculated the proportion of cases in both the birth cohort and hospital surveillance cohort that showed different signs of malnutrition throughout the study and have included these results within the manuscript text and in S1 Table.

Line 250 – 253: During home visits, between 18.4% and 30.8% of infants recorded a WHZ consistent with diagnoses of moderate to severe wasting, while 0.0% to 9.4% of reported HAZ and 2.4% to 12.8% of reported WAZ were in-line with moderate to severe stunting and underweight, respectively (S1 Table) [3].

Introduction: Both the introduction and discussion sections include the rationale that this pilot study forms the basis for larger studies by defining “challenges and workarounds” for similar studies in this community. However, the manuscript lacks an honest discussion of the challenges faced by this study, the considerations of future studies, and potential mitigation strategies for the challenges faced. For example, the retention rate in this birth cohort was very low and the authors do not discuss possible methods future studies can use to increase retention in this community. Further, lack of any stool sample in 25% of participants could pose a problem for future studies – what factors led to low attainment of specimens in this study and how can those be mitigated in the future? This manuscript would substantially benefit from a inclusion of a frank discussion about the challenges of this study.

We have taken this suggestion on board and have highlighted the challenges faced and suggested some mitigation strategies for the suggested focus areas.

Line 406 – 444: This study highlighted some of the significant research challenges faced in research in small LMIC settings and has several limitations. We had reasonably high attrition of our birth cohort, due to highly mobile families especially during the COVID-19 pandemic when the study was interrupted between April and July 2020 and during flash flooding events. This led to substantial loss to follow up and results that were under-powered to examine some important relationships. To increase study retention, our future study will increase focus on engagement and collaboration with participants and incorporate flexible and frequent follow-up methods. In particular, we will confirm whether the address provided at the hospital is a permanent or temporary residence (e.g., short-term stay with family closer to hospital than permanent residence) and maintain regular contact to update participants should delays occur between visits. Stool samples were not always available for collection at the time of visit and required return visits by the study team. Collecting specimens is an imposition on busy families, can be easily forgotten, requires training in safe handling, and provision of safety equipment. In the future, we will consider using flocked rectal swabs by trained study team members to collect specimens during home visits. These swabs have been relatively successful compares with traditional stool specimens for pathogen detection rates and bacterial culture [4, 5].

As we recruited all eligible children in hospital with a SAM or severe diarrhoea diagnosis until the target was met, the hospital cohort is likely not representative of children with these conditions in Dili. A future study would attempt to combat this by setting in place more specific demographic targets and include a control population of children diagnosed with other health conditions. We used

visual assessment of stool samples by laboratory staff to define diarrhoea in children. We note that visual inspection at the laboratory cannot provide details on the number of bowel movements, total stool volume, or duration of illness, nor can it compare the current sample to normal stool patterns in the infant [6]. Multiplex PCR testing is very sensitive and detects presence of pathogen nucleic acid but not necessarily viable pathogen. The Biofire GI PCR panel has shown high sensitivity and specificity in detection of diarrhoeal pathogens [7]. We intended to culture all human clinical *Salmonella, Campylobacter* and *Shigella* samples at NHL, however due to a lack of available resources this was not completed in most cases. Further, limited animal faecal or environmental samples were able to be collected following a positive PCR or culture result due an outbreak in African Swine fever in the first six months of the study. This limited our ability to estimate the prevalence of enteric infections in the surrounding home environment. In the absence of culture results to compare to the positive PCR results for *Campylobacter, Shigella* and *Salmonella* we were unable to determine the validity of the PCR results in this setting. However, similar studies have shown that multiplex PCR testing can provide an accurate description of enteric pathogen prevalence in low- and middle-income countries [8, 9]. This was pilot study to test acceptance and practicality of research and to build in-country laboratory and research capacity. Due to the funding available we were limited to recruiting from the main referral hospital and its immediate catchment area. Infants born at home or in provincial hospitals were not able to be included which may have led to bias in recruitment of urban or semi-urban families.

Methods: You need to add more details on the sample collection and laboratory methods. Can you name the 22 pathogens biofire looks for in the methods, most readers (and reviewers!) will not know. You should also discuss how the samples were processed, were they analyzed in real time, at what temperatures were they shipped and store?

In line with these and additional suggestions from reviewer 1, we have included further details on PCR methods. We have named all the pathogens screened using the BioFire GI panel, and have included details on sample preparation, media used, and other important aspects from the standard protocol.

Line 177 – 193: Laboratory staff processed samples from the birth cohort and hospital surveillance cohort using the same method. The NHL received faecal samples and performed visual inspections of the stool, routine microscopy including motility, and Gram stain morphology. In line with the standardised protocol, samples were stored either at room temperature (15-25°C) or refrigerated (2-8°C) for up to four days [1]. Laboratory staff transferred approximately 1g of stool to a screw-cap tube filled with 15ml of Cary Blair liquid medium (Remel, Lenexa, KS, USA) which was shaken until homogenous [2]. The NHL used the BioFire® Gastrointestinal Panel (GI) Kit for in-house PCR testing on all faecal samples from birth cohort and hospitalised children against 22 gastrointestinal pathogens, namely *Campylobacter* (*C. jejuni*/*C. coli*/*C. upsaliensis*), *Clostridioides* (*Clostridium*) *difficile* (toxin A/B), *Plesiomonas shigelloides*, *Salmonella*, *Yersinia enterocolitica*, *Vibrio* (*V. parahaemolyticus*/*V. vulnificus*/*V. cholerae*), Enteroaggregative *E. coli* (EAEC), Enteropathogenic *E. coli* (EPEC), Enterotoxigenic *E. coli* (ETEC) lt/st, Shiga-like toxin-producing *E. coli* (STEC) stx1/stx2, *E. coli* O157, *Shigella*/Enteroinvasive *E. coli* (EIEC), Adenovirus F40/41, Astrovirus, Norovirus GI/GII, Rotavirus A, Sapovirus (I, II, IV, and V), *Cryptosporidium*, *Cyclospora cayetanensis, Entamoeba histolytica*, and *Giardia lamblia*. Samples were prepared using a 30-test kit (RFIT-ASY-0116, bioMérieux Australia PTY LTD, AU) according to manufacturer's instructions [1]. We considered samples with a positive PCR result for two or more of the screened pathogens to have multiple pathogens detected.

Methods, line 159, what is "opportunistic sampling"? Does this mean that your sample is not representative? You should be far more precise with this term. Something like "we attempted to

recruit every eligible child that presented during working hours". And/or you need to acknowledge that your sample may not be representative in the limitations.

We have changed this sampling method description in line with these suggestions. Further, we have added a caveat in our limitations section of our discussion that explains how this sampling approach may not be representative of the population.

Line 166 – 167: Our team attempted to recruit every eligible child that presented to the hospital until we reached the target sample size.

Line 422 – 425: As we recruited all eligible children in hospital with a SAM or severe diarrhoea diagnosis until the target was met, the hospital cohort is likely not representative of children with these conditions in Dili. A future study would attempt to combat this by setting in place more specific demographic targets and include a control population of children diagnosed with other health conditions.

Results: You say that you recruited 19 children with diarrhea, but then only 16 are in the tables. This difference is never explained. You need to account for all the children in your study.

The difference in these numbers is due to the number of children recruited with a diagnosis of severe diarrhoea versus those with a submitted stool sample. We have added explanation of this different for clarity.

Line 300 – 302: Hospital doctors diagnosed 140 (88.1%, 95% CI 81.7–92.5%) children with SAM (132 with stool samples submitted to the NHL) and 19 (11.9%, 95% CI 7.5–18.3%) with severe diarrhoeal disease (13 with DWSD, and six with dysentery, 16 with stool samples submitted to the NHL).

Discussion: The discussion section of the manuscript focuses heavily on One Health and the need for future research on human-animal interaction. Additionally, lines 359-362 present challenges surrounding the collection of animal faecal and environmental samples. However, the One Health framework and environmental testing were not discussed at any other point within the manuscript. It is not included as a study aim, nor is environmental testing presented in the results section.

In line with reviewer 1's comment as well, we have re-included our methods and results on animal sampling. We have also explicitly stated our One Health aims in the introduction. We conducted limited animal sampling due to an African Swine Fever outbreak that occurred in the middle of our study. Where samples were collected, the animal health team successfully cultured three *Campylobacter* isolates. With this context within our manuscript, our suggestions for further collaboration are now supported.

Line 116 – 117: iii) identify household, environmental, and One Health risk factors associated with enteric pathogen infections to identify potential intervention targets.

Line 193 – 196: Samples positive by BioFire® PCR for selected enteric bacteria (Campylobacter, Salmonella and Shigella/EIEC) were reflex cultured on appropriate selective media and an animal health team returned to the household to collect animal samples.

Line 283 – 285: The animal health team collected and cultured three animal samples from PCR-positive *Campylobacter* human samples from households with chickens and/or pigs. All three samples were culture-positive for *Campylobacter*.

Discussion: The conclusion of the paper draw on essentially no information derived from the actual research conducted. As noted above, the first sentence of the conclusion references lesson learnt

without actually saying what those learnings were. The rest of the conclusion is more of an editorial comment that could have been written without conducting this pilot. I think the conclusion of the abstract is better. The authors need to tone down the editorializing and ensure the conclusion are drawn from their results.
In line with comments from reviewer 1, we have rewritten our conclusion to provide relevant outcomes and summaries in line with our data rather than in the general literature.

Line 445 – 454: We detected high prevalence of enteric pathogens, particularly the bacterial pathogens DEC and *Campylobacter* spp., and a high proportion of wasting from a young age in children in Dili, Timor-Leste. While we faced local challenges, we have identified strategies for mitigating these issues in the future and have produced practical information to guide the development of the next phase of this study. We suggest further exploration of risk factors in a high-powered study with a focus on feeding practices (e.g., exclusive bottle feeding) and the risk of enteric pathogen infection. Further, we recommend greater sampling from animal and environmental sources to identify potential transmission pathways. This would utilise a One Health collaborative approach between hospital and human health laboratory professionals and animal health experts including veterinarians and laboratory specialists to enhance local skills and capacity.

Minor Revisions
Abstract, methodology: “a pilot investigation of enteric disease…”, you really only look at pathogens which I think it is unfair to characterize as representative of the full spectrum on pediatric enteric disease. I would change to “enteropathogen prevalence”.

We have updated this first paragraph of our abstract methodology in line with reviewer 2’s suggestion.

Line 34 – 40: In our pilot study of enteric pathogens and malnutrition in Dili, Timor-Leste (July 2019–October 2020), we recruited 60 infants in a birth cohort from Hospital Nacional Guido Valadares (HNGV) with up to four home visits. We collected faecal samples and details of demographics, anthropometrics, diet and food practices, and animal husbandry. Additionally, we collected faecal samples, diagnostics, and anthropometrics from 160 children admitted to HNGV with a clinical diagnosis of severe diarrhoea or severe acute malnutrition (SAM). We tested faeces using the BioFire® FilmArray® Gastrointestinal Panel.

Abstract line 48: Bottle feeding compared to what? Breast feeding or family foods?

We have added the comparison group to this sentence.

Line 47 – 49: We associated bottle feeding with increased odds of pathogen detection when compared with exclusive breastfeeding at home (OR 8.3, 95%CI 1.1-62.7).

The authors appear to use the words “malnourished” and “stunted” imprecisely. I’d request the authors clarify specifically which definition of malnutrition the aims and results relate to. My assumption is that the authors mean to use just “stunted” (WHZ<-2) but include the terminology “malnourished or stunted” in their first aim without defining what they mean by “malnourished”. I suggest using specific and consistent language throughout.

Thank you for pointing out this error. We have changed this sentence in the aims to clarify that we mean measures of malnourishment including stunting, wasting and underweight. We have also updated throughout the manuscript, including the title.

Line 1 – 3: A pilot study using hospital surveillance and a birth cohort to investigate enteric pathogens and malnutrition in children, Dili, Timor-Leste.

Line 34 – 36: In our pilot study of enteric pathogens and malnutrition in Dili, Timor-Leste (July 2019–October 2020), we recruited 60 infants in a birth cohort from Hospital Nacional Guido Valadares (HNGV) with up to four home visits.

Line 60 – 61: This pilot study investigated the relationship between enteric disease and malnutrition in children in Dili, Timor-Leste.

Line 85 – 87: Such enteric pathogens potentially involved in malnutrition include enterotoxigenic *Escherichia coli* (ETEC), *Shigella,* and *Campylobacter* species [10-12].

Line 92 – 93: Recent studies have further implicated *Campylobacter* in child malnutrition.

Line 108 – 110: With the birth cohort, we aimed to i) estimate the proportion of moderately to severely malnourished children (with z-scores of ≤-2 for stunting, wasting, or underweight) during the first 12 months of life…

Line 119 – 120: This investigation formed the basis of a larger study to understand the impact of enteric pathogens on malnutrition.

Line 131 indicates that COVID-19 lockdowns delayed the 7- and 12-month visits for children in the birth cohort; however, the authors do not state how delayed these visits were nor do they describe at what age each visit is able to be conducted (can a 6-month old child have a 7-month visit, or a 14-month old child have a 12-month visit?). This could impact the understanding of the results shown about pathogen detection by age.

We have provided more detail on the modifications made to the home visit schedule in our methods. The mean and standard deviation for infant age at each visit is also presented in S1 Table. One standard deviation was within 2-3 weeks of the mean for each visit.

Line 133 – 136: Home visits were initially scheduled at two-month intervals from birth until six months of age. However, due to local flooding and COVID-19 lockdowns, visits occurred when infants were approximately one, four, seven, and 12 months of age (S1 Table).

Lines 305-309 discuss the lack of any significant relationships between pathogens and wasting or diarrheal disease. The authors conclude that this may indicate more complex multi-pathogen interactions or environmental factors, whereas it seems to me that the lack of significant findings may be solely due to the small sample size in this study. This rationale should be considered in the discussion section before concluding that any other factors are responsible for the results.

Thank you for this observation. We have changed our interpretation to include that the small sample size is the main factor for this lack of significant relationship. As the relationship between malnutrition and diarrhoeal disease is complex, we have included the additional explanations but have only included them as alternative/additional explanations.

Line 351 – 356: Despite high prevalence of certain pathogens, we did not detect any significant relationships between individual pathogens and wasting or diarrhoeal disease presentation. This is highly likely due to the small sample size of our study but could also be related to multiple pathogen interactions or additional nutritional or environmental factors. Pathogen virulence may also play an important part in the distribution of pathogens between malnourished and/or diarrhoeal children versus those that are relatively healthy [13].

You need to add a limitation that notes laboratory defined diarrhea has low sensitivity and often preforms poorly in young infants. See Levine GA (2017) Defining Pediatric Diarrhea in Low-Resource Settings

We have included this limitation in our discussion. Thank you for providing the additional reference to support this.

Line 425 – 429: We used visual assessment of stool samples by laboratory staff to define diarrhoea in children. We note that visual inspection at the laboratory cannot provide details on the number of bowel movements, total stool volume, or duration of illness, nor can it compare the current sample to normal stool patterns in the infant [6].

Additional Note: The authors state that all data are available within the manuscript; however, I do not see any data files beyond the tables and figures presented with the results.

We have included 1 supplementary figure, 1 supplementary note file and 7 supplementary tables in our PLOS One submission. We are confident that these additional files have provided all relevant information for reviewers.

Reviewer 3

The authors have conducted an important pilot survey of enteric pathogens in a region that is understudied in this respect. Their findings demonstrate a high prevalence of enteric pathogens and multiparasitism in the region, as well as a significant proportion of children with wasting already in the first year of life, SAM and DWSD, which indicates a population with need for study and health interventions. The results are in line with other large cohort studies. Although the authors make it clear that certain data was made much more difficult to collect due to the COVID-19 pandemic and outbreak of African Swine fever in the region, it is commendable that they were able to finish it and extrapolate useful data.

The paper needs a flowchart of recruited --> final group analyzed pathway showing how many were excluded in each group and for what reasons and at what point. As it is, the information is present in the paper, but scattered and therefore not as easy to track.

We have included a figure to visualise this flow chart of participant recruitment and analysis (Fig1).

Line 254 – 256: **Fig 1. Birth cohort recruitment and polymerase chain reaction (PCR) results in 60 infants, Dili, Timor-Leste, 2019–2020.** *See Table 1 for details on pathogens detected; +ve = positive, -ve = negative, LFTU = loss to follow up.

The authors have largely addressed the comments of the previous reviewers and made appropriate amendments.

Line 28-29: Provide a bit more background. In what way? How big is the problem? Nutrition-pathogen-EED cycle?

We have included more background in the abstract of this manuscript, in line with these suggestions.

Line 28 – 32: In low-to-middle-income countries (LMICs), enteric pathogens contribute to child malnutrition, affecting nutrient absorption, inducing inflammation, and causing diarrhoea. This is a substantial problem in LMICs due to high disease burden, poor sanitation and nutritional status, and the cyclical nature of pathogen infection and malnutrition. This relationship remains understudied in Timor-Leste.

Line 124: Why was only a single child of multiple births included?

We originally included this exclusion criteria as collecting information on two infants within the same household would not provide any additional benefit or information to the study, as we focused on household rather than individual level risk factors. We did not contact any multiple birth families and as such we have removed this sentence from the manuscript.

Line 131: Since the visits were delayed, does that mean it was still a 7-month and 12-month visit or did the ages changes at that point?

In line with these and additional queries from reviewer 2, we have provided more detail on the impact of COVID-19 and what these delays mean in relation to the original study protocol.

Line 133 – 136: Home visits were initially scheduled at two-month intervals from birth until six months of age. However, due to local flooding and COVID-19 lockdowns, visits occurred when infants were approximately one, four, seven, and 12 months of age (S1 Table).

Line 145-151: The conditions for fecal storage until processing have not been specified, please insert. What does "correctly collected and stored" mean? Are there cold chain/preservative requirements?

We have included further detail in the methods on storage protocols and procedures. Stool samples remained at room temperature but out of direct sunlight until they were transferred to the insulated containers. We have also provided details on laboratory storage of the pathogen, including temperature requirements.

Line 154 – 157: Parents and/or guardians were provided with instructions in their native language, Tetum, to ensure samples were collected on the day of or the evening before the home visit and stored in a cool location prior to transport. Our team provided appropriate equipment including gloves, spatula, specimen container, and clinical waste bag.

Line 179 – 181: In line with the standardised protocol, samples were stored either at room temperature (15-25°C) or refrigerated (2-8°C) for up to four days (22).

Line 171: Reference # 22 only leads to a link for the commercial assay used, it does not lead to a description of a method. Please either provide a link the method or describe it.

In line with these and additional comments from reviewer 1 and 2, we have provided further detail on the PCR methods including explaining in full the pathogens screen using BioFire. We have also included a link to the standard BioFire GI Panel protocol.

Line 177 – 192: Laboratory staff processed samples from the birth cohort and hospital surveillance cohort using the same method. The NHL received faecal samples and performed visual inspections of the stool, routine microscopy including motility, and Gram stain morphology. In line with the standardised protocol, samples were stored either at room temperature (15-25°C) or refrigerated (2-8°C) for up to four days [1]. Laboratory staff transferred approximately 1g of stool to a screw-cap tube filled with 15ml of Cary Blair liquid medium (Remel, Lenexa, KS, USA) which was shaken until homogenous [2]. The NHL used the BioFire® Gastrointestinal Panel (GI) Kit for in-house PCR testing on all faecal samples from birth cohort and hospitalised children against 22 gastrointestinal pathogens, namely *Campylobacter* (*C. jejuni*/*C. coli*/*C. upsaliensis*), *Clostridioides* (*Clostridium*) *difficile* (toxin A/B), *Plesiomonas shigelloides*, *Salmonella*, *Yersinia enterocolitica*, *Vibrio* (*V. parahaemolyticus*/*V. vulnificus*/*V. cholerae*), Enteroaggregative *E. coli* (EAEC), Enteropathogenic *E. coli* (EPEC), Enterotoxigenic *E. coli* (ETEC) lt/st, Shiga-like toxin-producing *E. coli* (STEC) stx1/stx2, *E. coli* O157, *Shigella*/Enteroinvasive *E. coli* (EIEC), Adenovirus F40/41, Astrovirus, Norovirus GI/GII, Rotavirus A, Sapovirus (I, II, IV, and V), *Cryptosporidium*, *Cyclospora cayetanensis, Entamoeba*

histolytica, and *Giardia lamblia*. Samples were prepared using a 30-test kit (RFIT-ASY-0116, bioMérieux Australia PTY LTD, AU) according to manufacturer's instructions [1].

Line 183: Specify between which groups.

We have specified which groups we compared in different analyses.

Line 210 – 211: We performed Fisher's exact and Chi-square tests to analyse differences between pathogen group (bacteria, viruses, or parasites), age group, and home visit.

Line 226 – 228: We performed Fisher's exact, Chi-square, and Student's t-Test, where appropriate, to analyse differences between pathogens detected by age group and hospital diagnosis.

Line 226-227: "There was a weak relationship between…" should be backed by some statistical measure. Either there is no relationship or a trend but with no significance or a significant correlation. Looking at Fig 2 visually, there does not appear to be a relationship except maybe at 7 months but stats would be important to determine the strength of relationship.

We have updated this graph to use the raw number of pathogens detected per sample against WHZ in Fig 3 (old Fig 2) and conducted linear regression calculations for each visit to test the relationship of the line. Visits one, two, and four didn't return any significant results, but in line with reviewer 3's interpretation, visit three showed a significant negative trend between number of pathogens and WHZ. We have noted this in our results text, also highlighting the limits on interpreting the results because of the small sample size and wide confidence intervals.

Line 267 – 271: While we did not detect a significant relationship between WHZ and pathogens detected for visits one, two, and four (linear model p value >0.05), we did detect a significant negative trend in visit three (p value <0.01). However, we note that the small sample size and wide confidence intervals limit the interpretation of these results (Fig 2).

Lines 305-309: It could also be that the study is a bit underpowered to detect these differences, considering how many pathogens are being measured.

Thank you for this observation. We have addressed this in a similar comment from reviewer 2. Line numbers of the changed text are provided below.

Line 351 – 356: Despite high prevalence of certain pathogens, we did not detect any significant relationships between individual pathogens and wasting or diarrhoeal disease presentation. This is highly likely due to the small sample size of our study but could also be related to multiple pathogen interactions or additional nutritional or environmental factors. Pathogen virulence may also play an important part in the distribution of pathogens between malnourished and/or diarrhoeal children versus those that are relatively healthy [13].

Line 337: I very much like this point in the discussion. Quite a few studies have failed to find association between a single risk factor like preparation of food, waste disposal type, probably because it can be difficult to truly establish that relationship. But environmental sampling could inform the general exposure risk to the pathogens and perhaps even point to more specific pathogen sources in the particular sampling sites (important because they are likely to vary site to site). Even in the SHINE study, which had an incredibly extensive multi-factorial WASH intervention, it was difficult to reduce pathogen exposure and the authors concluded that likely larger infrastructure interventions are required to reduce overall pathogen exposure, which one could measure with environmental sampling.

Thank you for this insight and findings from a previous study. We are unable to provide any detailed conclusions from this pilot study but will include environmental sampling in the next stage of this important project and will consider the conclusions from the SHINE study when interpreting these data.

In the supplementary tables in general, mention in the caption that the star indicates statistical significance.

We have updated the captions to include this information and have checked through the supplementary tables for consistency.

Line 638 – 650 and 655 – 658: **S2 Table. Adjusted univariate odds ratios using a generalised estimating equations model for differences in pathogens detected between diarrhoeal and non-diarrhoea stool samples for infants in a birth cohort in Dili, Timor-Leste, 2019–2020.** * Indicates statistical significance (p value <0.05). 95% CI = 95% confidence interval. ref = reference variable. NA = odds ratio not calculated.

S3 Table. Adjusted univariate odds ratios using a generalised estimating equations model for risk factors associated with pathogen detection for infants in a birth cohort, Dili, Timor-Leste, 2019-2020. * Indicates statistical significance (*p* value <0.05). GEE aOR = adjusted odds ratio from generalised estimating equation model. 95% CI = 95% confidence interval. ref = reference variable. NA = odds ratio not calculated.

S4 Table. Adjusted univariate odds ratios using a generalised estimating equations model for risk factors associated with moderate wasting for infants in a birth cohort, Dili, Timor-Leste, 2019–2020. * Indicates statistical significance (*p* value <0.05). GEE aOR = adjusted odds ratio from generalised estimating equation model. WHZ = weight-for-height z-score. 95% CI = 95% confidence interval. ref = reference variable. NA = odds ratio not calculated.

S7 Table. Adjusted univariate odds ratios using a generalised linear model for differences in pathogens detected between diarrhoeal and non-diarrhoea stool samples for children in a hospital surveillance cohort in Dili, Timor-Leste, 2019–2020. * Indicates statistical significance (p value <0.05). aOR = adjusted odds ratio. 95% CI = 95% confidence interval. ref = reference variable. NA = odds ratio not calculated.

The discussion should expand a bit on the significant finding of exclusive bottle-feeding and increased risk of pathogens in stools, especially since it confirms findings from a previous smaller study in Timor-Leste (different district). https://doi.org/10.1177/1010539513486175

Thank you for this suggestion and reference. We have included a further discussion of early cessation of breast feeding and animal risks in our discussion.

Line 375 – 384: We investigated risk factors in the home and surrounding environment in the birth cohort. We found that exclusive bottle feeding had an increased odds of pathogen detection compared to breast feeding. This is in line with a previous study in the Aileu District of Timor-Leste where early cessation of exclusive breastfeeding led to increased risk of exposure to contaminated water and food and unsatisfactory hygiene practices with subsequent diarrhoeal illness and malnutrition [14]. Further, although we did not find any significant animal-related risk factors, we were able to culture *Campylobacter* from animal stool in households where a human sample tested positive. Contact with animals and animal stool in the home environment could be an important transmission pathway for *Campylobacter* infections in children, with dogs and hens known to track human faecal material from open defecation into the home [14].

Suggestions for the future studies the authors have planned

Although it is unorthodox to include suggestions for future studies, this was only a pilot study and the authors plan to expand on this important topic. Hence, a couple of suggestions below:

- You can keep track of the specific month of birth in order to make correlations to pathogens, anthropometrics etc. with rainfall, availability of crops etc. as potential mediating variables
- In case you plan to expand on the number of risk factors measured and if you have enough participants, you could also consider clustering methods for studying the effects of aggregate variable on a single outcome such as pathogen-positive stools or recovery from SAM. Sorry this is a paper for studying soccer players but it nicely describes some potential techniques where the number of variables are in the 10s: https://link.springer.com/article/10.1007/s10479-023-05185-w#Sec2
- Considering what this pilot study was for, and the data you were able to collect, I would agree that focusing on WHZ as your primary anthropometric makes sense. However, if you plan to have a longer and larger cohort, I would absolutely include analysis for the other two major metrics, as well as mean upper arm circumference and I would also run some predictive models on those.
- Sometimes it can be difficult to see relationships to the anthropometrics when they're just set as a binary, especially with WHZ which is more dynamic than LAZ. Two additional approaches to try are: 1) use regression models where the outcome is the continuous WHZ variable rather than the binary 2) Defining a group that is "persistently" wasting (so across several consecutive time-points).

We thank the reviewer for their time and effort with these future study suggestions. We have not included any further details of these in the current manuscript but will take these into consideration for our next stage of studies in Timor-Leste.

Reviewer 4

Child malnutrition has been one of the most prioritized public health issues in Timor-Leste. As previous nationwide representative data suggested that diarrhea was associated with child malnutrition, focusing on relationships between child malnutrition and detected enteric pathogens should be meaningful. However, it does not seem that the article showed valuable findings effectively.

General comments
Title:
The title is redundant. Stunting is part of malnutrition. In addition, "investigation" is an unclear term, and it does not tell the audience what is studied in this paper.

We have updated our study title to be clearer and more informative.

Line 1 – 3: A pilot study using hospital surveillance and a birth cohort to investigate enteric pathogens and malnutrition in children, Dili, Timor-Leste.

Introduction:
The target population is not well defined. Although only limited to children born in the HNGV, the birth cohort may be representative, at least partially, of children in Dili. But children from hospital surveillance did not represent the general population. Even SAM cases and severe diarrhea cases

must have different groups. I believe birth cohort data, SAM cases, and severe diarrhea cases should not be combined to perform statistical analyses. The "population" where the sample was drawn should be considered in statistical analyses.

We have included details of the representativeness of the hospital cohort in our limitations section of the discussion. Further, we have suggested some strategies to overcome this issue in future studies. We have disaggregated the combined birth cohort and hospitalised cohort diarrhoeal stool analysis and have conducted these again separately. These results are presented in supplementary tables S2 and S7 and are presented in our results text. We did not detect any statistical differences between diarrhoeal and non-diarrhoeal samples in the birth cohort, and while we did detect differences in *Salmonella* and *Shigella* detection in the hospitalised cohort, the small sample size and wide confidence intervals limit the interpretation of these results.

Line 422 – 425: As we recruited all eligible children in hospital with a SAM or severe diarrhoea diagnosis until the target was met, the hospital cohort is likely not representative of children with these conditions in Dili. A future study would attempt to combat this by setting in place more specific demographic targets and include a control population of children diagnosed with other health conditions.

Line 280 – 281: We did not detect any significant differences in pathogens detected based on visually assessed diarrhoeal versus non-diarrhoeal stool samples (S2 Table).

Line 318 – 321: We detected more PCR-positive *Salmonella* (aOR 17.2, 95% CI 1.6–201.3) and *Shigella* (aOR 3.8, 95% CI 1.0–14.4) in visually assessed diarrhoeal stool samples compared with non-diarrhoeal samples but note that the small sample size and wide confidence intervals limit the interpretation of these results (S7 Table).

Line 638 – 641: **S2 Table. Adjusted univariate odds ratios using a generalised estimating equations model for differences in pathogens detected between diarrhoeal and non-diarrhoea stool samples for infants in a birth cohort in Dili, Timor-Leste, 2019–2020.** * Indicates statistical significance (p value <0.05). 95% CI = 95% confidence interval. ref = reference variable. NA = odds ratio not calculated.

Line 655 – 658: **S7 Table. Adjusted univariate odds ratios using a generalised linear model for differences in pathogens detected between diarrhoeal and non-diarrhoea stool samples for children in a hospital surveillance cohort in Dili, Timor-Leste, 2019–2020.** * Indicates statistical significance (p value <0.05). aOR = adjusted odds ratio. 95% CI = 95% confidence interval. ref = reference variable. NA = odds ratio not calculated.

In my understanding, there were three research purposes. They might be:
- To estimate child malnutrition's prevalence and calculate Z-scores according to children's age.
- To estimate the prevalence of children with enteric pathogens, to count the number of pathogens detected per each child, and to identify types of detected pathogens.
- To identify risk factors of pathogen detection and wasting.

These could have been done by birth cohort data, even though the sample size might be small.

I do not think authors' research purpose iii) is valid because different populations were mixed. I suggest hospital surveillance data be analyzed differently in a separate paper with clearly mentioned research purposes and methods. Or, if authors wish to combine two sub-projects into one article, it is suggested to identify clearly stated objectives for each dataset and to organize methods and results accordingly.

We have revised our study aims in line with these suggestions. We have clearly defined the purpose of each study aim and have separated each aim by study cohort. The first three aims relate to the birth cohort, and the final two relate to the hospitalised cohort. To clarify reviewer 4's comment above about the validity of the analysis for risk factors, this analysis was only conducted on the birth cohort – populations were not mixed. We have ensured that the methods and results are also clearly laid out by study group and are ordered according to study aim order, where appropriate.

Line 111 – 122: With the birth cohort, we aimed to i) estimate the proportion of moderately to severely malnourished children (with z-scores of ≤-2 for stunting, wasting, or underweight) during the first 12 months of life, ii) determine the prevalence of enteric pathogens in stool samples using polymerase chain reaction (PCR), identify those with multiple pathogen detections, identify the most common pathogens detected, and identify any differences in pathogen detections between diarrhoeal and non-diarrhoeal stool, and iii) identify household, environmental, and One Health risk factors associated with enteric pathogen infections to identify potential intervention targets. For the hospital surveillance cohort, we aimed to iv) quantify malnourishment using different metrics in children diagnosed with severe acute malnutrition (SAM) or severe diarrhoea, and v) estimate the prevalence of enteric pathogens in stool samples, identify those with multiple pathogen detections, identify the most common pathogens, and identify any differences in pathogen detections between diarrhoeal and non-diarrhoeal stool in children admitted with these conditions.

Methods, Results, and Discussion
Please re-write and organize Methods, Results, and Discussion sections based on the revised research objectives with appropriate subtitles.

As above, we have reorganised our study sections in line with our revised study aims. We have included appropriate subheadings relating to each cohort for clarity throughout.

In particular, please carefully select tables shown in the main manuscript and put in supplemental files based on the revised research purposes.

We have reviewed our included tables and figures in the main manuscript and supplementary materials. We are confident that these are correctly placed.

Specific comments
- Lines 224-227 are difficult to read. Figure 2 is complicated, and it does not clearly show difference in pathogens detected by age in months, difference in Z-scores by age in months, and difference in Z-scores by pathogens detected.

We have rewritten this section of the results to be clearer. Additionally, we have recreated Fig 2 (now Fig 3) to provide clearer relationships between WHZ and number of pathogens detected for each visit. We were also able to calculate whether these relationships were significant using a linear model and have written about this in the results section. We note that the small sample size limits the interpretation of these results.

Line 264 – 271: We observed significant differences in pathogen detection across home visits. As visits progressed, both the number of pathogens detected within the cohort and the number of multiple pathogen detections in individual infants generally increased (p <0.05; **Error! Reference source not found.** and S1 Fig). While we did not detect a significant relationship between WHZ and pathogens detected for visits one, two, and four (linear model p value >0.05), we did detect a significant negative trend in visit three (p value <0.01). However, we note that the small sample size and wide confidence intervals limit the interpretation of these results (Fig 2).

Line 275 – 276: **Fig 2 . Linear regression model for weight-for-height Z-score by number of pathogens detected for each birth cohort visit, Dili, Timor-Leste, 2019–2020.** 95% confidence intervals are indicated in grey shading for each line.

- Lines 377-382 are not based on the research findings. The paper does not findings from animal data.

We have now included our animal testing results and a small discussion section within the manuscript text, which provides a basis for this claim in our conclusion. We have rewritten this conclusion in line with other reviewers' suggestions.

Line 283 – 285: The animal health team collected and cultured three animal samples from PCR-positive Campylobacter human samples from households with chickens and/or pigs. All three samples were culture-positive for Campylobacter.

Line 379 – 384: Further, although we did not find any significant animal-related risk factors, we were able to culture *Campylobacter* from animal stool in households where a human sample tested positive. Contact with animals and animal stool in the home environment could be an important transmission pathway for *Campylobacter* infections in children, with dogs and hens known to track human faecal material from open defecation into the home [14].

Yours sincerely,

Danielle Cribb
