# Supplementary material for: A pilot study using hospital surveillance and a birth cohort to investigate enteric pathogens and malnutrition in children, Dili, Timor-Leste
Source: PLoS One. 2024 Feb 1;19(2):e0296774. doi: 10.1371/journal.pone.0296774 (PMC10833528; doi:10.1371/journal.pone.0296774)
Supplement: Attachment — Submitted filename: PONE-D-23-14515 reviews of original submission.pdf [file pone.0296774.s014.pdf]

Date: Jul 12 2023 08:54PM
To: "Danielle M. Cribb" danielle.cribb@anu.edu.au
From: "PLOS ONE" plosone@plos.org
Subject: PLOS ONE Decision: Revision required [PONE-D-23-14515]
Attachment(s): PONE-D-23-14515 Reviewer Comments.pdf

PONE-D-23-14515
A pilot investigation of enteric pathogens and stunting and malnutrition using a combination of hospital surveillance and a birth cohort of children in Dili, Timor-Leste.
PLOS ONE

Dear Dr. Cribb,

Thank you for submitting your manuscript to PLOS ONE. After careful consideration, we feel that it has merit but does not fully meet PLOS ONE's publication criteria as it currently stands. Therefore, we invite you to submit a revised version of the manuscript that addresses the points raised during the review process.

Please submit your revised manuscript by Aug 26 2023 11:59PM. If you will need more time than this to complete your revisions, please reply to this message or contact the journal office at plosone@plos.org. When you're ready to submit your revision, log on to https://www.editorialmanager.com/pone/ and select the 'Submissions Needing Revision' folder to locate your manuscript file.

Please include the following items when submitting your revised manuscript:

- A rebuttal letter that responds to each point raised by the academic editor and reviewer(s). You should upload this letter as a separate file labeled 'Response to Reviewers'.
- A marked-up copy of your manuscript that highlights changes made to the original version. You should upload this as a separate file labeled 'Revised Manuscript with Track Changes'.
- An unmarked version of your revised paper without tracked changes. You should upload this as a separate file labeled 'Manuscript'.

If you would like to make changes to your financial disclosure, please include your updated statement in your cover letter. Guidelines for resubmitting your figure files are available below the reviewer comments at the end of this letter.

If applicable, we recommend that you deposit your laboratory protocols in protocols.io to enhance the reproducibility of your results. Protocols.io assigns your protocol its own identifier (DOI) so that it can be cited independently in the future. For instructions see: https://journals.plos.org/plosone/s/submission-guidelines#loc-laboratory-protocols. Additionally, PLOS ONE offers an option for publishing peer-reviewed Lab Protocol articles, which describe protocols hosted on protocols.io. Read more information on sharing protocols at https://plos.org/protocols?utm_medium=editorial-email&utm_source=authorletters&utm_campaign=protocols.

We look forward to receiving your revised manuscript.

Kind regards,

José António Baptista Machado Soares, PhD
Academic Editor
PLOS ONE

Journal requirements:

When submitting your revision, we need you to address these additional requirements.

1. Please ensure that your manuscript meets PLOS ONE's style requirements, including those for file naming. The PLOS ONE style templates can be found at

https://journals.plos.org/plosone/s/file?id=wjVg/PLOSOne_formatting_sample_main_body.pdf and

https://journals.plos.org/plosone/s/file?id=ba62/PLOSOne_formatting_sample_title_authors_affiliations.pdf

2. Please include a complete copy of PLOS' questionnaire on inclusivity in global research in your revised manuscript. Our policy for research in this area aims to improve transparency in the reporting of research performed outside of researchers' own country or community. The policy applies to researchers who have travelled to a different country to conduct research, research with Indigenous populations or their lands, and research on cultural artefacts. The questionnaire can also be requested at the journal's discretion for any other submissions, even if these conditions are not met.

Please find more information on the policy and a link to download a blank copy of the questionnaire here: https://journals.plos.org/plosone/s/best-practices-in-research-reporting. Please upload a completed version of your

questionnaire as Supporting Information when you resubmit your manuscript.

Additional Editor Comments:

Dear authors,
I am pleased to say that most of the reviewers enjoyed the manuscript very much and we are excited about the possibility to publish your work. However, several different concerns were raised by them, and I agreed with them. For example, the study presented a lot of data in every possible manner with no focus on the actual focus on the actual objective of the study. The conclusion of the paper draws on essentially no information derived from the actual research conducted. The authors state that all data are available within the manuscript; however, reviewers do not see any data files beyond the tables and figures presented with the results. Another concern is how the data is shown and it would be recommended to check the statistical analyses. Please answer careful all the comments and concerns of the reviewers. See their comments below.
So, I kindly invite the authors to realize a thoughtful revision of the submitted version to achieve the improvement asked by the Reviewer and Plos ONE journal.

Thank you and best regards,
António Machado

[Note: HTML markup is below. Please do not edit.]

Reviewers' comments:

Reviewer's Responses to Questions

Comments to the Author

1. Is the manuscript technically sound, and do the data support the conclusions?

The manuscript must describe a technically sound piece of scientific research with data that supports the conclusions. Experiments must have been conducted rigorously, with appropriate controls, replication, and sample sizes. The conclusions must be drawn appropriately based on the data presented.

Reviewer #1: Partly

Reviewer #2: Partly

Reviewer #3: Yes

Reviewer #4: Partly

---

2. Has the statistical analysis been performed appropriately and rigorously?

Reviewer #1: No

Reviewer #2: Yes

Reviewer #3: Yes

Reviewer #4: No

---

3. Have the authors made all data underlying the findings in their manuscript fully available?

The PLOS Data policy requires authors to make all data underlying the findings described in their manuscript fully available without restriction, with rare exception (please refer to the Data Availability Statement in the manuscript PDF file). The data should be provided as part of the manuscript or its supporting information, or deposited to a public repository. For example, in addition to summary statistics, the data points behind means, medians and variance measures should be available. If there are restrictions on publicly sharing data—e.g. participant privacy or use of data from a third party—those must be specified.

Reviewer #1: No

Reviewer #2: No

Reviewer #3: Yes

Reviewer #4: Yes

---

4. Is the manuscript presented in an intelligible fashion and written in standard English?

PLOS ONE does not copyedit accepted manuscripts, so the language in submitted articles must be clear, correct, and

unambiguous. Any typographical or grammatical errors should be corrected at revision, so please note any specific errors here.

Reviewer #1: No

Reviewer #2: Yes

Reviewer #3: Yes

Reviewer #4: Yes

---

5. Review Comments to the Author

Please use the space provided to explain your answers to the questions above. You may also include additional comments for the author, including concerns about dual publication, research ethics, or publication ethics. (Please upload your review as an attachment if it exceeds 20,000 characters)

Reviewer #1: Comments:-
The authors present their work regarding enteric pathogens and malnutrition in Timor-Leste and expected to find out the risk factors associated with them. However; the study presented a lot of data in every possible manner with no focus on the actual focus on the actual objective of the study. Since its a pilot study, it should also collect some significant number of fecal samples from community settings. The number of sample size is not adequate to come to a conclusion.

Q 1. a BioFire® PCR assay was performed to test all faecal samples-How PCR was performed, since it is a multiplex technique, how many primers and samples were loaded at a time?
No methodology for PCR was mentioned, no images and results for PCR was shared, please share
Was it performed in-house or outsourced?
Q 2. When you mention that 63.9% isolates were positive for multiple pathogens (how many numbers you consider to call it multiple pathogens}?
3. Please write no of total samples in tables above
4. Methodology is confusing and appears to be non-repetitive
5. Results are misleading as different numbers are mentioned at different places
6. Risk factors and their associations with enteric pathogens and malnutrition in Timor-Leste is not explored as mentioned in the conclusion.
7. Massive English language writing skills are required as it is full of grammatical and typo errors.
8. Methodology and results showed few lines for PCR but the discussion is just on PCR, which was not the main aim of the study.
9. One Health collaborative approach is a good idea to work on this study but it was merely pointed as a suggestion
10. The study started with a lot of promises and data to be explored but concluded randomly with no definite outcome and conclusion.

Reviewer #2: This pilot study aimed to understand the landscape of malnutrition and enteric pathogens among children in Dili, Timor-Leste. The authors rationalize, and I agree, that this study has the potential to provide important framework for the development of larger studies aimed at understanding risk factors and outcomes of malnutrition and enteric pathogens among children in this community. The authors have clearly worked hard on this study, and it does offer helpful information about enteropathogens in a region of the world that is a bit of data blindspot. However, to have the utility that the authors hope for, this manuscript needs some major revisions, in addition to the changes made in response to the previous round of reviews.

Major Revisions
Introduction: In the paragraph beginning at line 102, the authors introduce the 3 aims of this study. Aim 1 states that they planned to "estimate the proportion of children from a birth cohort who were malnourished or stunted at any time during the first 12 months of life". However, I do not see any results in the manuscript that address this aim. The authors share the average z-scores among children in the cohort, but do not present a proportion of birth cohort children with malnutrition or wasting in the 12-month period.

Introduction: Both the introduction and discussion sections include the rationale that this pilot study forms the basis for larger studies by defining "challenges and workarounds" for similar studies in this community. However, the manuscript lacks an honest discussion of the challenges faced by this study, the considerations of future studies, and potential mitigation strategies for the challenges faced. For example, the retention rate in this birth cohort was very low and the authors do not discuss possible methods future studies can use to increase retention in this community. Further, lack of any stool sample in 25% of participants could pose a problem for future studies – what factors led to low attainment of specimens in this study and how can those be mitigated in the future? This manuscript would substantially benefit from a inclusion of a frank discussion about the challenges of this study.

Methods: You need to add more details on the sample collection and laboratory methods. Can you name the 22 pathogens biofire looks for in the methods, most readers (and reviewers!) will not know. You should also discuss how the samples were processed, were they analyzed in real time, at what temperatures were they shipped and store?

Methods, line 159, what is "opportunistic sampling"? Does this mean that your sample is not representative? You should be far more precise with this term. Something like "we attempted to recruit every eligible child that presented during working hours". And/or you need to acknowledge that your sample may not be representative in the limitations.

Results: You say that you recruited 19 children with diarrhea, but then only 16 are in the tables. This difference is never explained. You need to account for all the children in your study.

Discussion: The discussion section of the manuscript focuses heavily on One Health and the need for future research on human-animal interaction. Additionally, lines 359-362 present challenges surrounding the collection of animal faecal and environmental samples. However, the One Health framework and environmental testing were not discussed at any other point within the manuscript. It is not included as a study aim, nor is environmental testing presented in the results section.

Discussion: The conclusion of the paper draw on essentially no information derived from the actual research conducted. As noted above, the first sentence of the conclusion references lesson learnt without actually saying what those learnings were. The rest of the conclusion is more of an editorial comment that could have been written without conducting this pilot. I think the conclusion of the abstract is better. The authors need to tone down the editorializing and ensure the conclusion are drawn from their results.

Minor Revisions
Abstract, methodology: "a pilot investigation of enteric disease…", you really only look at pathogens which I think it is unfair to characterize as representative of the full spectrum on pediatric enteric disease. I would change to "enteropathogen prevalence".

Abstract line 48: Bottle feeding compared to what? Breast feeding or family foods?

The authors appear to use the words "malnourished" and "stunted" imprecisely. I'd request the authors clarify specifically which definition of malnutrition the aims and results relate to. My assumption is that the authors mean to use just "stunted" (WHZ<-2) but include the terminology "malnourished or stunted" in their first aim without defining what they mean by "malnourished". I suggest using specific and consistent language throughout.

Line 131 indicates that COVID-19 lockdowns delayed the 7- and 12-month visits for children in the birth cohort; however, the authors do not state how delayed these visits were nor do they describe at what age each visit is able to be conducted (can a 6-month old child have a 7-month visit, or a 14-month old child have a 12-month visit?). This could impact the understanding of the results shown about pathogen detection by age.

Lines 305-309 discuss the lack of any significant relationships between pathogens and wasting or diarrheal disease. The authors conclude that this may indicate more complex multi-pathogen interactions or environmental factors, whereas it seems to me that the lack of significant findings may be solely due to the small sample size in this study. This rationale should be considered in the discussion section before concluding that any other factors are responsible for the results.
You need to add a limitation that notes laboratory defined diarrhea has low sensitivity and often preforms poorly in young infants. See Levine GA (2017) Defining Pediatric Diarrhea in Low-Resource Settings
Additional Note: The authors state that all data are available within the manuscript; however, I do not see any data files beyond the tables and figures presented with the results.

Reviewer #3: Main Comments
The authors have conducted an important pilot survey of enteric pathogens in a region that is understudied in this respect. Their findings demonstrate a high prevalence of enteric pathogens and multiparasitism in the region, as well as a significant proportion of children with wasting already in the first year of life, SAM and DWSD, which indicates a population with need for study and health interventions. The results are in line with other large cohort studies. Although the authors make it clear that certain data was made much more difficult to collect due to the COVID-19 pandemic and outbreak of African Swine fever in the region, it is commendable that they were able to finish it and extrapolate useful data.
The paper needs a flowchart of recruited --> final group analyzed pathway showing how many were excluded in each group and for what reasons and at what point. As it is, the information is present in the paper, but scattered and therefore not as easy to track.
The authors have largely addressed the comments of the previous reviewers and made appropriate amendments.
Please see the attached file to view and address specific remarks.

Reviewer #4: Child malnutrition has been one of the most prioritized public health issues in Timor-Leste. As previous nationwide representative data suggested that diarrhea was associated with child malnutrition, focusing on relationships between child malnutrition and detected enteric pathogens should be meaningful. However, it does not seem that the article showed valuable findings effectively.

General comments
Title:
The title is redundant. Stunting is part of malnutrition. In addition, "investigation" is an unclear term, and it does not tell the audience what is studied in this paper.

Introduction:
The target population is not well defined. Although only limited to children born in the HNGV, the birth cohort may be representative, at least partially, of children in Dili. But children from hospital surveillance did not represent the general population. Even SAM cases and severe diarrhea cases must have different groups. I believe birth cohort data, SAM cases, and severe diarrhea cases should not be combined to perform statistical analyses. The "population" where the sample was drawn should be considered in statistical analyses.

In my understanding, there were three research purposes. They might be:
- To estimate child malnutrition's prevalence and calculate Z-scores according to children's age.
- To estimate the prevalence of children with enteric pathogens, to count the number of pathogens detected per each child, and to identify types of detected pathogens.
- To identify risk factors of pathogen detection and wasting.
These could have been done by birth cohort data, even though the sample size might be small.

I do not think authors' research purpose iii) is valid because different populations were mixed. I suggest hospital surveillance data be analyzed differently in a separate paper with clearly mentioned research purposes and methods. Or, if authors wish to combine two sub-projects into one article, it is suggested to identify clearly stated objectives for each dataset and to organize methods and results accordingly.

Methods, Results, and Discussion
Please re-write and organize Methods, Results, and Discussion sections based on the revised research objectives with appropriate subtitles.

In particular, please carefully select tables shown in the main manuscript and put in supplemental files based on the revised research purposes.

Specific comments
- Lines 224-227 are difficult to read. Figure 2 is complicated, and it does not clearly show difference in pathogens detected by age in months, difference in Z-scores by age in months, and difference in Z-scores by pathogens detected.
- Lines 377-382 are not based on the research findings. The paper does not findings from animal data.

---

6. PLOS authors have the option to publish the peer review history of their article (what does this mean?). If published, this will include your full peer review and any attached files.

If you choose "no", your identity will remain anonymous but your review may still be made public.

Do you want your identity to be public for this peer review? For information about this choice, including consent withdrawal, please see our Privacy Policy.

Reviewer #1: No

Reviewer #2: No

Reviewer #3: **Yes:** Gordana Panic

Reviewer #4: No

[NOTE: If reviewer comments were submitted as an attachment file, they will be attached to this email and accessible via the submission site. Please log into your account, locate the manuscript record, and check for the action link "View Attachments". If this link does not appear, there are no attachment files.]

While revising your submission, please upload your figure files to the Preflight Analysis and Conversion Engine (PACE) digital diagnostic tool, https://pacev2.apexcovantage.com/. PACE helps ensure that figures meet PLOS requirements. To use PACE, you must first register as a user. Registration is free. Then, login and navigate to the UPLOAD tab, where you will find detailed instructions on how to use the tool. If you encounter any issues or have any questions when using PACE, please email PLOS at figures@plos.org. Please note that Supporting Information files do not need this step.

In compliance with data protection regulations, you may request that we remove your personal registration details at any time. (Remove my information/details). Please contact the publication office if you have any questions.
