# Supplementary material for: A pilot study using hospital surveillance and a birth cohort to investigate enteric pathogens and malnutrition in children, Dili, Timor-Leste
Source: PLoS One. 2024 Feb 1;19(2):e0296774. doi: 10.1371/journal.pone.0296774 (PMC10833528; doi:10.1371/journal.pone.0296774)
Supplement: Attachment — Submitted filename: Response to reviewers.pdf [file pone.0296774.s016.pdf]

13/12/2023

Dear Editor,

We thank you and the reviewers for your comments on our manuscript entitled “A pilot study using hospital surveillance and a birth cohort to investigate enteric pathogens and malnutrition in children, Dili, Timor-Leste.”. We have carefully reviewed these comments and have amended the manuscript accordingly. We have provided a point-by-point response below. Reviewer comments are in black text with our responses in blue text.

In addition, we have ensured that file names and manuscript text meet PLOS ONE’s style requirements and have corrected the grant information in ‘Funding Information’ section.

Reviewer #1

I would now recommend publication with minor adjustments (see below). The authors have demonstrated a staggering prevalence of pathogens and malnutrition for the critically understudied Dili Timor-Leste region, and these numbers should be published. The authors have accomplished what one usually sets out to accomplish in pilot studies: though underpowered (as pilot surveys often are), they have provided a foundational evidence base upon which to inform future studies in this area. This is critical for informing local and national governments on areas of focus for research and intervention.

I would echo the statements of some reviewers that it should be heavily emphasized that the study is not sufficiently powered to demonstrate likely true non-associations and that only future larger studies can do this. This is predominantly a survey but a survey is the foundational evidence one gathers to demonstrate a need for larger studies.

Response: we have reiterated that the study is underpowered throughout the manuscript

Line 241: However, we note that the small sample size and wide confidence intervals limit the interpretation of these results (Fig 3).

Line 264: We note that the study is not sufficiently powered to demonstrate true non-associations.

Line 287-291: We detected more PCR-positive Salmonella (aOR 17.2, 95% CI 1.6–201.3) and Shigella (aOR 3.8, 95% CI 1.0–14.4) in visually assessed diarrhoeal stool samples compared with non-diarrhoeal samples but we note that the small sample size and wide confidence intervals limit the interpretation of these results (S7 Table).

Line 326-328: This is most likely due to the small sample size of our study but could also be related to multiple pathogen interactions or additional nutritional or environmental factors.

Line 382-383: This led to substantial loss to follow up and results that were under-powered to examine some important relationships.

I would disagree with one reviewer that the two cohorts should be two separate papers. First, the two individual papers would be too small individually. If the study was larger, I would agree, but this is a small pilot study. Second, the results were analyzed separately, which is the right approach, and the two cohorts complement one another in that one sees that pathogens associated with hospitalization may not necessarily be the same ones associated with malnutrition in the community.

Response: thank you for this comment. We agree that the two studies should remain within the same paper but as separate analyses.

Abstract: This relationship remains ~~understudiesin~~ understudied in Timor-Leste.

Response: We have corrected this error in the manuscript:

Line 24-25: This relationship remains understudied in Timor-Leste.

Line 182: account for repeated observations with the R package "geepack", where appropriate [30]. GEE enables

Response: we have corrected this error in the manuscript to include an 's' after GEE throughout.

Line 180-184: We calculated odds ratios (OR), controlling for age, sex, and season, using generalised estimating equations (GEEs) to account for repeated observations with the R package "geepack", where appropriate [30]. GEEs enable effective control for repeated measures by incorporating within-subject or within-cluster correlations, thereby providing robust analysis of longitudinal or clustered data [31].

Line 238: has an extra blank space after "p<0.05;"

Response: we have corrected this error in the manuscript.

Line 239: … infants generally increased (p <0.05; Table 1 and S1 Fig).

Line 242: has an extra blank space after "Fig 3"

Response: we have corrected this error in the manuscript.

Line 241-242: However, we note that the small sample size and wide confidence intervals limit the interpretation of these results (Fig 3).

Line 330: "Most pathogens were recovered from faecal samples with limited or no evidence of diarrhoeal disease upon visual assessment". Do you mean in the birth cohort or hospital or both?

Response: we mean both cohorts. We have updated the text to clarify this.

Line 335-336: Most pathogens were recovered from faecal samples with limited or no evidence of diarrhoeal disease upon visual assessment in both study arms.

Line 503: For the reference used to provide more information on the PCR protocol, add the link and access date: https://www.biofiredx.com/e-labeling/ITI0030

Response: thank you for providing this reference. We have included this in our reference list.

Line 508-509: bioMérieux. BioFire® FilmArray® Gastrointestinal (GI) Panel (RFIT-ASY-0104). Available from: https://www.biofiredx.com/e-labeling/ITI0030. Accessed 13 December 2023.

We have also updated other web resources with an access date – we have provided the most recent access date to confirm the resource's availability on lines 512, 514, 518, 522, 536, 538-539.

I was intrigued by the relatively low rate of rotavirus and I wondered if that is due to high vaccination coverage for rotavirus in this region? Since it is typically a source of diarrheal disease and malnutrition in the tropics, I thought the authors could comment on this in the discussion.

Response: thank you for raising this interesting point. We have included a brief discussion on rotavirus prevalence, vaccine introduction, and coverage in our discussion, supported by relevant references.

Line 322-327: We detected low prevalence of rotavirus in infants and children in both study arms. Routine vaccination for rotavirus commenced in Timor-Leste in late 2019, coinciding with our study [47]. While coverage rates are not reported for 2019, estimates from 2021 indicate approximately 80% coverage [48]. A study in Thai children receiving rotavirus vaccines found that the burden of diarrhoeal-related disease decreased after the rotavirus vaccine was introduced [49].

Line 587-597:

47. World Health Organization. Vaccination schedule for Timor-Leste. Accessed from: https://immunizationdata.who.int/pages/schedule-by-country/tls.html. Accessed on 13 December 2023.

48. Timor-Leste: WHO and UNICEF estimates of immunization coverage: 2022 revision. Data received as of June 26, 2023. Accessed from: https://data.unicef.org/wp-content/uploads/cp/immunisation/tls.pdf. Accessed 13 December 2023.

49. Charoenwat, B., Suwannaying, K., Paibool, W. et al. The impact of rotavirus vaccination on acute diarrhea in Thai children under 5 years of age in the first year of universal implementation of rotavirus vaccines in the National Immunization Program (NIP) in Thailand: a 6-year analysis. BMC Public Health 23, 2109 (2023). https://doi.org/10.1186/s12889-023-16958-0

Reviewer #2

Excellent work. It is a good idea to raise it as a pilot study. Ah futuro se le puede dar diferentes direccions con el proposito de aprovecharlo al máximo. My suggestions point to the visualization of the study. I would like to have a graphical abstract and add and/or combine some graphs into one.

Thank you for your comments. We have combined figures 2a, 2b and 2c into a single image file with 'A', 'B', and 'C' above each graph. We have created a graphical abstract for the manuscript and have included this as a supplementary figure (S2 Fig), referenced within the discussion section on line 310.

Yours sincerely,

Danielle Cribb
