# Supplementary material for: A pilot study using hospital surveillance and a birth cohort to investigate enteric pathogens and malnutrition in children, Dili, Timor-Leste
Source: PLoS One. 2024 Feb 1;19(2):e0296774. doi: 10.1371/journal.pone.0296774 (PMC10833528; doi:10.1371/journal.pone.0296774)
Supplement: Attachment — Submitted filename: Reviewer Comments for PONE-D23-35940.pdf [file pone.0296774.s013.pdf]

Reviewer Comments for PONE-D-23-35940

I would now recommend publication with minor adjustments (see below). The authors have demonstrated a staggering prevalence of pathogens and malnutrition for the critically understudied Dili Timor-Leste region, and these numbers should be published. The authors have accomplished what one usually sets out to accomplish in pilot studies: though underpowered (as pilot surveys often are), they have provided a foundational evidence base upon which to inform future studies in this area. This is critical for informing local and national governments on areas of focus for research and intervention.

Major Comments

I would echo the statements of some reviewers that it should be heavily emphasized that the study is not sufficiently powered to demonstrate likely true non-associations and that only future larger studies can do this. This is predominantly a survey but a survey is the foundational evidence one gathers to demonstrate a need for larger studies.

I would disagree with one reviewer that the two cohorts should be two separate papers. First, the two individual papers would be too small individually. If the study was larger, I would agree, but this is a small pilot study. Second, the results were analyzed separately, which is the right approach, and the two cohorts complement one another in that one sees that pathogens associated with hospitalization may not necessarily be the same ones associated with malnutrition in the community.

Minor Comments

Abstract: This relationship remains ~~understudiesin~~ understudied in Timor-Leste.

Line 182: account for repeated observations with the R package "geepack", where appropriate [30]. GEE enables

Line 238: has an extra blank space after "p<0.05;"

Line 242: has an extra blank space after "Fig 3"

Line 330: "Most pathogens were recovered from faecal samples with limited or no evidence of diarrhoeal disease upon visual assessment". Do you mean in the birth cohort or hospital or both?

Line 503: For the reference used to provide more information on the PCR protocol, add the link and access date: https://www.biofiredx.com/e-labeling/ITI0030

Additional Comments

I was intrigued by the relatively low rate of rotavirus and I wondered if that is due to high vaccination coverage for rotavirus in this region? Since it is typically a source of diarrheal disease and malnutrition in the tropics, I thought the authors could comment on this in the discussion.
