# Supplementary material for: A pilot study using hospital surveillance and a birth cohort to investigate enteric pathogens and malnutrition in children, Dili, Timor-Leste
Source: PLoS One. 2024 Feb 1;19(2):e0296774. doi: 10.1371/journal.pone.0296774 (PMC10833528; doi:10.1371/journal.pone.0296774)
Supplement: Attachment — Submitted filename: PONE-D-23-14515 Reviewer Comments.pdf [file pone.0296774.s015.pdf]

Reviewer Comments

Main Comments

The authors have conducted an important pilot survey of enteric pathogens in a region that is understudied in this respect. Their findings demonstrate a high prevalence of enteric pathogens and multiparasitism in the region, as well as a significant proportion of children with wasting already in the first year of life, SAM and DWSD, which indicates a population with need for study and health interventions. The results are in line with other large cohort studies. Although the authors make it clear that certain data was made much more difficult to collect due to the COVID-19 pandemic and outbreak of African Swine fever in the region, it is commendable that they were able to finish it and extrapolate useful data.

The paper needs a flowchart of recruited → final group analyzed pathway showing how many were excluded in each group and for what reasons and at what point. As it is, the information is present in the paper, but scattered and therefore not as easy to track.

The authors have largely addressed the comments of the previous reviewers and made appropriate amendments.

Minor comments

Line 28-29: Provide a bit more background. In what way? How big is the problem? Nutrition-pathogen-EED cycle?

Line 124: Why was only a single child of multiple births included?

Line 131: Since the visits were delayed, does that mean it was still a 7-month and 12-month visit or did the ages changes at that point?

Line 145-151: The conditions for fecal storage until processing have not been specified, please insert. What does "correctly collected and stored" mean? Are there cold chain/preservative requirements?

Line 171: Reference # 22 only leads to a link for the commercial assay used, it does not lead to a description of a method. Please either provide a link the method or describe it.

Line 183: Specify between which groups.

Line 226-227: “There was a weak relationship between…” should be backed by some statistical measure. Either there is no relationship or a trend but with no significance or a significant correlation. Looking at Fig 2 visually, there does not appear to be a relationship except *maybe* at 7 months but stats would be important to determine the strength of relationship.

Lines 305-309: It could also be that the study is a bit underpowered to detect these differences, considering how many pathogens are being measured.

Line 337: I very much like this point in the discussion. Quite a few studies have failed to find association between a single risk factor like preparation of food, waste disposal type, probably because it can be difficult to truly establish that relationship. But environmental sampling could inform the general exposure risk to the pathogens and perhaps even point to more specific pathogen sources in the particular sampling sites (important because they are likely to vary site to site). Even in the SHINE study, which had an incredibly extensive multi-factorial WASH intervention, it was difficult to reduce pathogen exposure and the authors concluded that likely larger infrastructure interventions are required to reduce overall pathogen exposure, which one could measure with environmental sampling.

In the supplementary tables in general, mention in the caption that the star indicates statistical significance.

The discussion should expand a bit on the significant finding of exclusive bottle-feeding and increased risk of pathogens in stools, especially since it confirms findings from a previous smaller study in Timor-Leste (different district). https://doi.org/10.1177/1010539513486175

Suggestions for the future studies the authors have planned

Although it is unorthodox to include suggestions for future studies, this was only a pilot study and the authors plan to expand on this important topic. Hence, a couple of suggestions below:

- You can keep track of the specific month of birth in order to make correlations to pathogens, anthropometrics etc. with rainfall, availability of crops etc. as potential mediating variables
- In case you plan to expand on the number of risk factors measured and if you have enough participants, you could also consider clustering methods for studying the effects of aggregate variable on a single outcome such as pathogen-positive stools or recovery

from SAM. Sorry this is a paper for studying soccer players but it nicely describes some potential techniques where the number of variables are in the 10s: https://link.springer.com/article/10.1007/s10479-023-05185-w#Sec2

- Considering what this pilot study was for, and the data you were able to collect, I would agree that focusing on WHZ as your primary anthropometric makes sense. However, if you plan to have a longer and larger cohort, I would absolutely include analysis for the other two major metrics, as well as mean upper arm circumference and I would also run some predictive models on those.
- Sometimes it can be difficult to see relationships to the anthropometrics when they're just set as a binary, especially with WHZ which is more dynamic than LAZ. Two additional approaches to try are: 1) use regression models where the outcome is the continuous WHZ variable rather than the binary 2) Defining a group that is "persistently" wasting (so across several consecutive time-points).
